# Separation of saccharides using fullerene-bonded silica monolithic columns via π interactions in liquid chromatography

**DOI:** 10.1038/s41598-020-70904-3

**Published:** 2020-08-14

**Authors:** Hiroshi Kobayashi, Kazuya Okada, Shinnosuke Tokuda, Eisuke Kanao, Yusuke Masuda, Toyohiro Naito, Hikaru Takaya, Mingdi Yan, Takuya Kubo, Koji Otsuka

**Affiliations:** 1 Shinwa Chemical Industries Ltd., 50-2, Kagekatsu-cho, Fushimi-ku, Kyoto, 612-8307 Japan; 2 Graduate School of Engineering, Kyoto University, Nishikyo-ku, Katsura, Kyoto, 615-8510 Japan; 3 Institute of Chemical Research, Kyoto University, Gokashou, Uji, Kyoto, 611-0011 Japan; 4 Department of Chemistry, University of Massachusetts Lowell, One University Ave., Lowell, MA 01854 USA

**Keywords:** Analytical chemistry, Organic chemistry, Surface chemistry, Theoretical chemistry, Nanoscale materials

## Abstract

We report on a potential method to separate sugars by using the specific interaction between fullerenes and saccharides in liquid chromatography (LC). Aromatic rings with high electron density are believed to interact strongly with saccharides due to CH–π and/or OH–π interactions. In this study, the fullerene-bonded columns were used to separate saccharides by LC under aqueous conditions. As a result, 2-aminobenzamide-labeled glucose homopolymer (Glcs) was effectively separated by both C60 and C70 columns in the range of Glc-1 to Glc-20 and high blood glucose level being retained in greater quantity. Furthermore, similar separations were identified by LC–mass spectrometry with non-labeled glucose homopolymers. Theoretical study based on molecular dynamics and DFT calculation demonstrated that a supramolecular complex of saccharide–fullerene was formed through CH–π and/or OH–π interactions, and that the interactions between saccharide and fullerene increase with the increase units of the saccharide. Additionally, the C60 column retained disaccharides containing maltose, trehalose, and sucrose. In this case, it was assumed that the retention rates were determined by the difference of the dipole moment in each saccharide. These results suggest that the dipole-induced dipole interaction was dominant, and that maltose—with the higher dipole moment—was more strongly retained compared to other disaccharides having lower dipole moment.

## Introduction

Recently, biopharmaceuticals have been widely applied in a variety of human medical therapies, and glycoprotein-based antibodies have performed remarkably well, dominating the more typical low molecular weight medicines. Biopharmaceuticals can have very individualized designs through the installation of specific ligands, and therefore can be used as efficient tumor-targeted drug delivery systems^1,2^. Generally, most of these biopharmaceuticals are produced by collecting antibodies from an immunized animal, such as a mouse. Heterogeneous sugars are then introduced during the process of post-translational modification. The difference of the sugar chains affects the activity of the antibodies^3,4^ and their stability^5,6^; therefore, the separation procedures used are key to ensure that the medicinal properties of the molecule are retained. These antibodies are usually separated by using liquid chromatographic techniques such as affinity chromatography^7–9^, size exclusion^10,11^, ion-exchange^12,13^, and hydrophobic interaction^14^ to remove contamination. However, the differences in the sugar chains cannot be identified using these techniques.

On the other hand, many methods for separating saccharides have been developed in liquid chromatography (LC). For example, boronate affinity has been utilized due to its ability to recognize diols and further applications have also been reported^15^. Additionally, ionic liquids^16^ and capillary electrophoresis^17^ have been recently applied to the separation of a variety of carbohydrates. Hydrophilic interaction chromatography (HILIC) is one of the greatest tools for the separation of saccharides in LC^18–21^. In HILIC, a hydration sphere onto the separation media provides specific interaction with polar compounds. Therefore, the interaction is interfered in case of using aqueous solvents with higher water contents as a mobile phase, and the solubility of most saccharides is not advantageous in organic solvents, which are usually employed in HILIC with higher contents as the mobile phases. As a unique separation system for saccharides, even in using aqueous solvents with higher water contents, porous graphite carbon (PGC) is often used, especially for the separation of sugar chains cleaved from glycoproteins^22–24^. In this system, the CH–π interaction based on the dipole-induced dipole interaction may contribute to the interaction between PGC and saccharides^25–27^. The potential energy of the dipole-induced dipole interaction can be expressed as an Eq. (1)^28^

1 $$ U = - \mu^{{2}} \alpha / \, ({\text{4p}}e_{0} e_{r} )^{{2}} r^{{6}} $$

where *U*, *μ*, *α*, *ε*_0_, *ε*_*r*_, and *r* are the stabilization energy, dipole moment of a polar molecule, polarizability of a stationary phase, dielectric constant in vacuum, dielectric constant of a solute, and intermolecular distance, respectively. Based on the theoretical understanding, we expect that a PGC with high polarizability provides the strongest dipole-induced dipole interaction. Furthermore, Zhao et al. reported the strength of the CH–π interaction between aromatic rings and alkyl chains by peak shifts in ^1^H-NMR, and subsequently demonstrated that larger number of electrons in aromatic rings and higher polarizability provided increase of the CH–π interaction^29,30^. Therefore, PGC with rich electrons may indicate a strong CH–π interaction with saccharides. Also, it is anticipated that each sugar chain has a different dipole moment, resulting in the difference of the CH–π interaction with PGC. Fernández-Alonso et al. indicated the difference in the CH–π interaction which occurs due to the position of the OH groups in monosaccharides by ^1^H-NMR and computer simulations^31,32^.

As well as PGC, nanocarbon materials such as fullerenes, are expected to provide effective π interactions due to their high electron density^33–36^. Previously, we successfully prepared C_60_- or C_70_-fullerene (C60, C70) bonded separation media using a silica monolithic capillary and a thermo-reactive agent, perfluorophenyl azide. Then, the specific π interactions were identified for spherical recognition^37–40^, CH–π interaction^41,42^, and halogen–π interaction^43,44^. According to these effective intermolecular recognitions, we anticipated that the fullerene-bonded separation media might be useful for the separation of saccharides via the CH– or OH–π interaction as well as dipole interaction. In this communication, we investigated the possibility of separating saccharides (Fig. S1) with fullerene-bonded columns in LC and used computer simulation to assess theoretical considerations.

## Results and discussion

**Figure 1:**
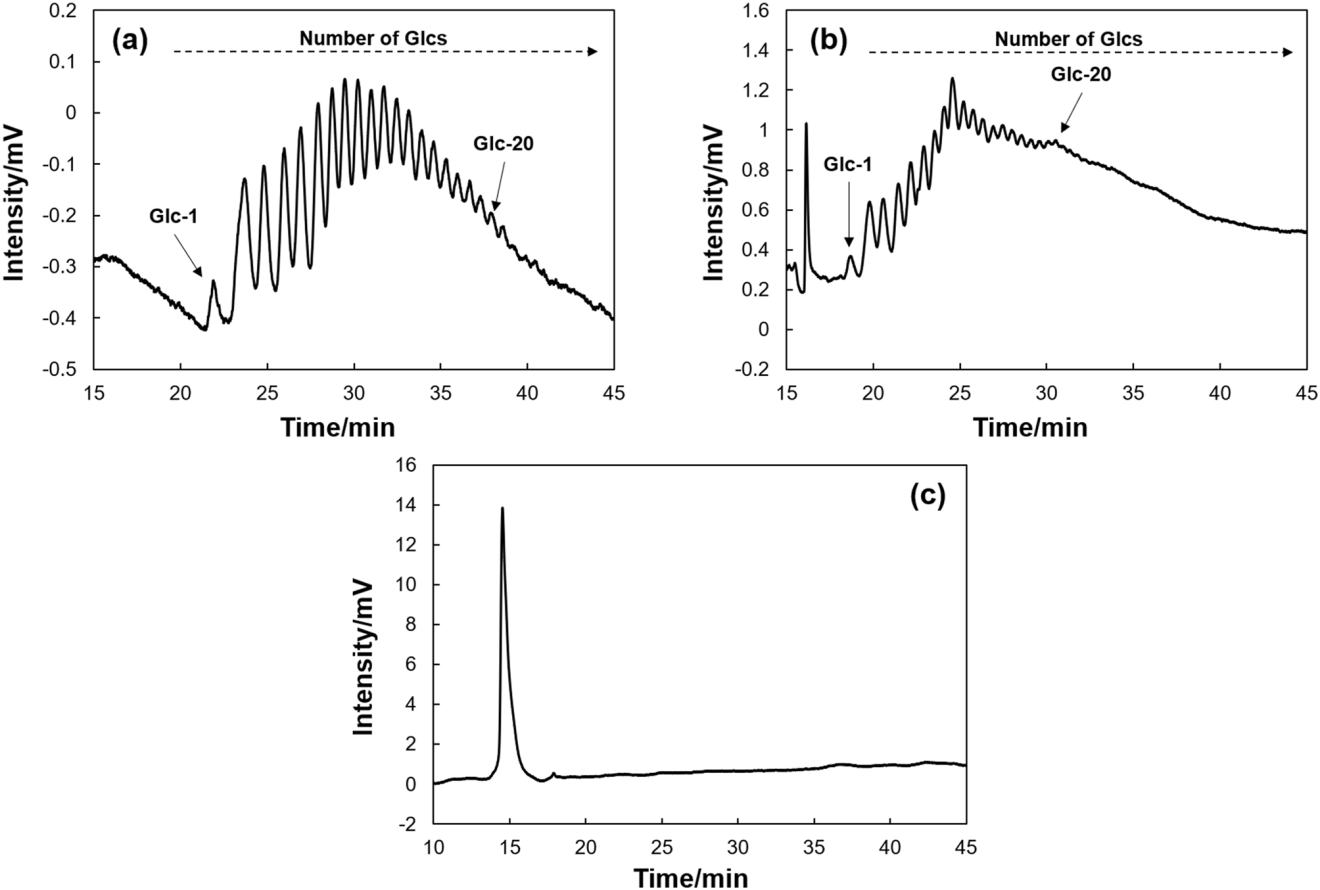
Chromatograms of 2AB-labeled Glcs in LC. LC conditions: columns, (**a**) C60, (**b**) C70, (**c**) ODS; column size, 25.0 cm × 100 μm i.d.; flow rate, 350 nL min^–1^; mobile phase, 2–4% 2-propanol aq. linear gradient for 45 min (C60 column), 4–8% 2-propanol aq. linear gradient for 45 min (C70, ODS column); detection, UV 214 nm.

C60- or C70-conjugated molecules were successfully synthesized using the methods of our previous studies^41,43^. Then, C60- or C70-bonded silica monolithic capillaries (C60 column or C70 column) were also prepared (see Scheme S1, S2, and Fig. S2). In general, most saccharides are not available for detection by UV absorption, so instead chromophores are utilized for this detection. Here, we employed 2-aminobenzamide (2-AB), which is well-known as a labelling reagent for saccharides^45^. In the separation of 2-AB labeled saccharides, even an authentic hydrophobic column modified with octadecyl-silyl groups (ODS) under gradient elution with aqueous acetonitrile (MeCN) worked for efficient separation as shown in Fig. S3. In this process, the difference in the hydrophobicity of the labeled saccharides contributed to the separation. As mentioned above, we anticipated that the π interactions could be worked between fullerenes and saccharides, therefore, MeCN was not suitable for the mobile phase because C≡N group might interfere the π interactions. Thus, we optimized the mobile phase conditions for the separation of 2-AB labeled glucose homopolymers (2AB-Glcs) using C60 and C70 columns in LC with the reversed-phase mode. As an additive for the gradient elution, 2-propanol was employed. Figure 1 shows the chromatograms of 2AB-Glcs in C60, C70, and ODS columns. From these chromatograms, we confirmed the efficient separations of 2AB-Glcs by using C60 and C70 columns. The elution order of 2AB-Glcs was identified based on an increase in retention along with increasing the number of glucose units, although the detections of high blood glucose level, especially over Glc-20 were difficult because of the lower relative UV intensity. In contrast, all 2AB-Glcs were eluted together in the ODS column. Interestingly, although the ODS column worked for the separation with the MeCN gradient (Fig. S3), the 2-propanol gradient did not affect separation. According to these separation behaviors, it is assumed that the separation mechanisms in the fullerene-bonded columns are affected not only by the hydrophobic interaction but also by CH–π and/or OH–π interactions between fullerenes and Glcs, as they were in our previous study^46^.

**Figure 2:**
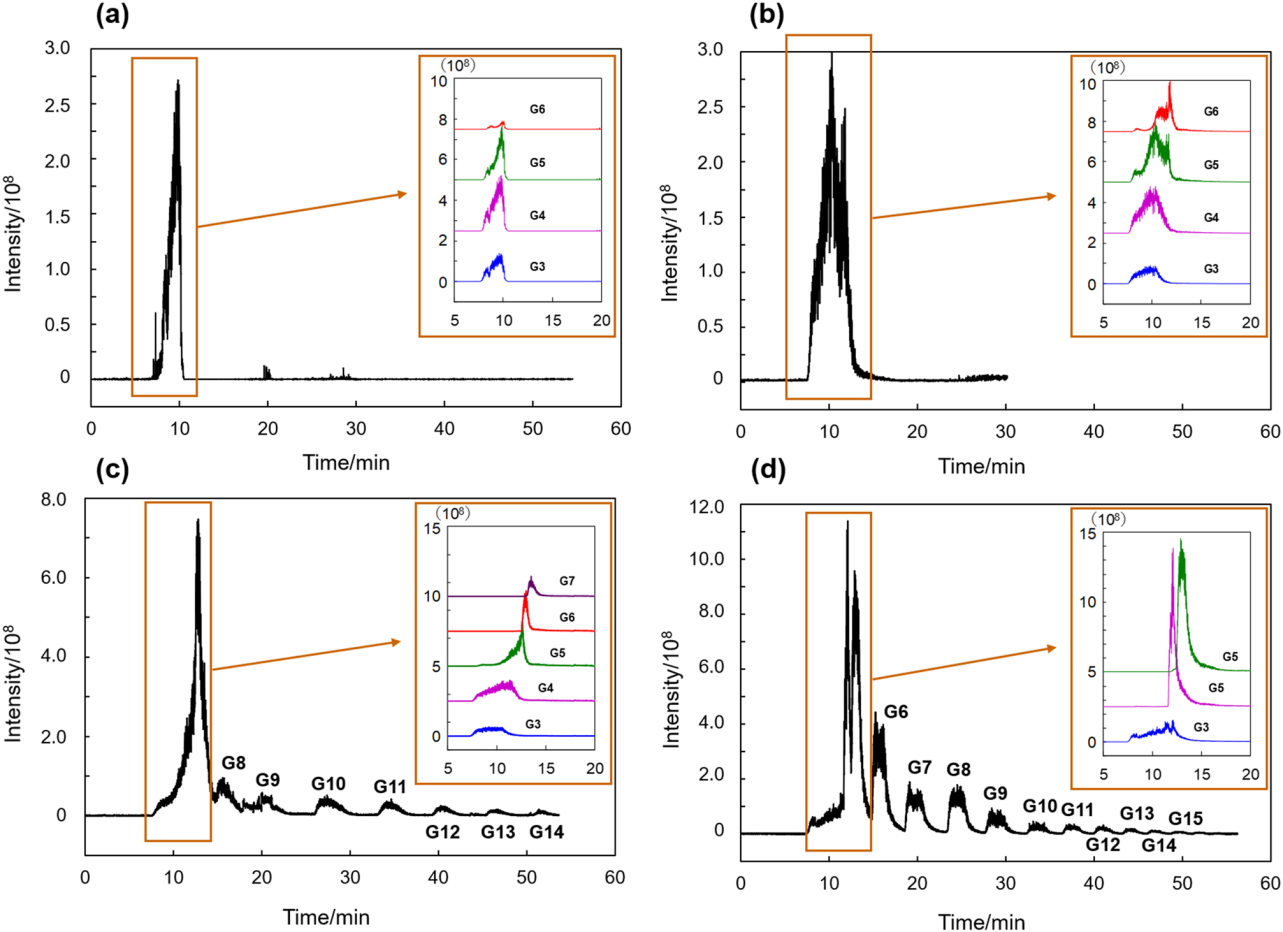
Chromatograms of non-labeled Glcs in LC–MS. Conditions: columns, (**a**) ODS (end-capping), (**b**) ODS (no-capping), (**c**) C60, and (**d**) C70; column size, 25. 0 cm × 100 μm i.d.; flow rate, 350 nL min^–1^; mobile phase, (**a**, **b**) 2–6% acetonitrile aq. linear gradient for 45 min, (**c**) 2–4% 2-propanol aq. linear gradient for 45 min, (**d**) 4–8% 2-propanol aq. linear gradient for 45 min; detection, negative ion mode.

To confirm the contribution of the π interactions between fullerenes and Glcs, non-labeled Glcs were analyzed by LC–mass spectrometry (LC–MS). Furthermore, ODS columns were also evaluated to compare the hydrophobic interaction and the effect of silanol groups. For each column, we employed the optimized LC conditions, in which the best separation of Glcs was attained. The total ion chromatogram (TIC) and the separated Glcs (selected molecular weight) of each homopolymer are summarized in Fig. 2. Briefly, to identify the effect of the remaining silanol groups in the silica-based columns^47^, the ODS column, which was end-capped with acetyl chloride (ODS_capp_), was also evaluated. As shown in Fig. 2a,b, the separation of Glcs was not observed in either ODS column. Although the retention of Glcs was slightly higher in the non-capped ODS column, both the hydrophobicity and the interaction with silanol groups did not affect the retentions for Glcs in the aqueous LC conditions. On the other hand, the C60 and C70 columns showed some separation of Glcs as shown in Fig. 2c,d. Compared to the separation of 2AB-labeled Glcs, the retention strengths of Glcs were weaker in non-labeled Glcs. The results are easily understood because of the hydrophobicity and π–π interaction among 2AB/fullerenes. Interestingly, the Glcs were still separated due to the increase of the glucose unit in both columns. Here, according to our previous studies, fullerenes effectively allowed π interactions, such as the CH–π and/or OH–π interactions^41,42,46^. For this comparison, the measurements were conducted for non-labelled saccharides with the applied columns and the results showed that the separation was achieved for using C60/C70 columns, but not for using C18 columns. Therefore, it would be better to say the significance/advantage of separation of non-labelled saccharides.

**Figure 3:**
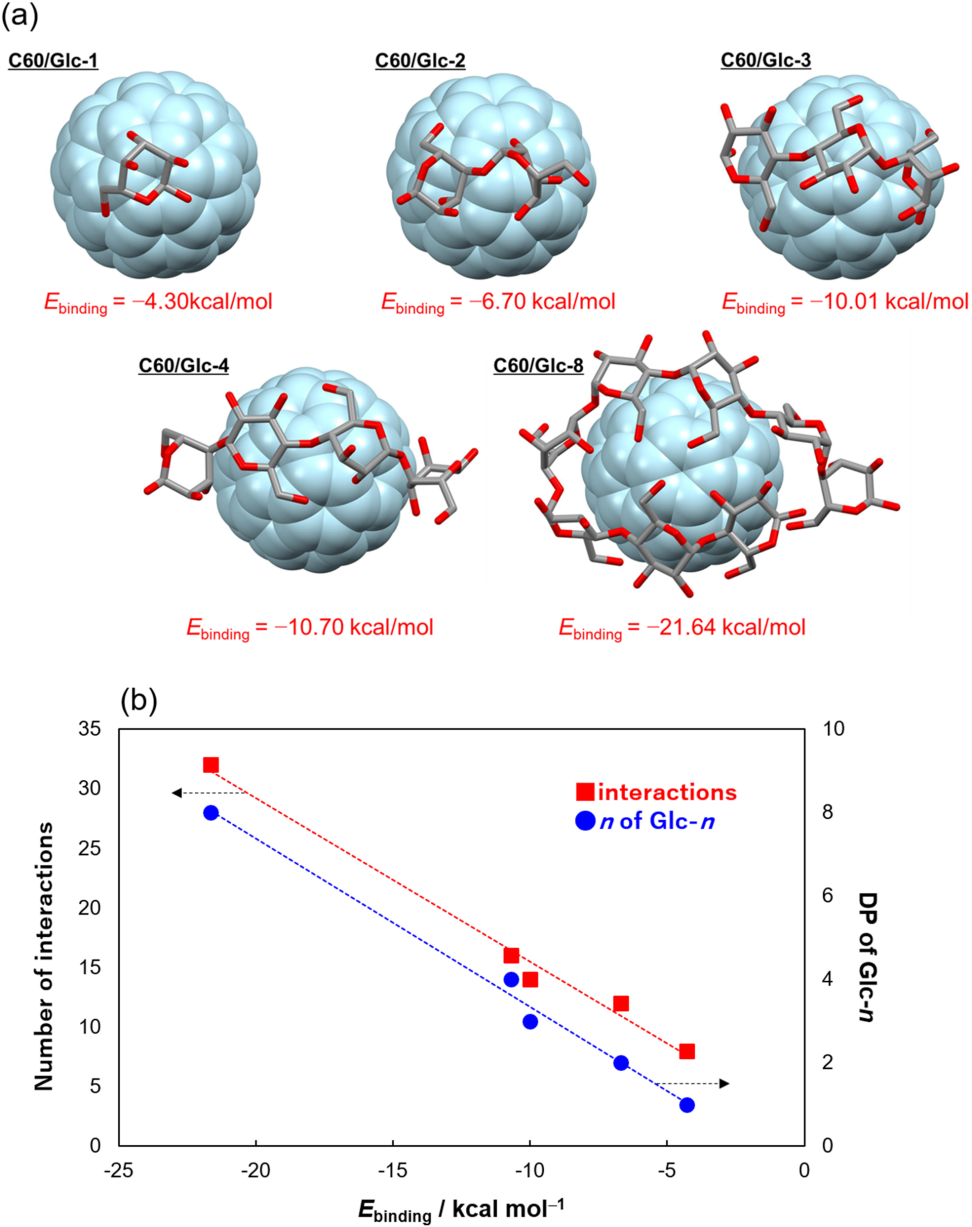
(**a**) Stabilized structure and the binding energy (*E*_binding_) among C60/Glc-*n* in water and (**b**) Relationships between the energy (*E*_binding_, kcal/mol) and the degree of polymerization of (Glcs)_n_ (n = 1–4, 8) and the total number of interactions including CH–π, OH–π, and O:–π.

The contribution of non-bonding interactions in the LC-separation of Glcs was assessed by the stabilization energy gained through the formation of fullerene C60/Glcs supramolecular complex. Theoretical simulations were carried out to calculate the stabilization energy (*E*_binding_) of C60/(Glc-*n*) with their supramolecular structures which reveal the precise binding mechanism between the aromatic surface of C60 and Glcs. The initial molecular geometry of C60/Glcs complex was explored by simulate annealing (SA) and the resulting most stable structure was further optimized by density functional theory (DFT) calculation at the M06-2x/6-31G(d,p) level of theory including a solvent effect of water to afford the stabilized structures of Glc-1, 2, 4, and 8 as shown in Fig. 3a and Figs. S4–S8.

The stabilization energy of *E*_binding_ was finally calculated on these structures by removing a basis set superposition error (BSSE)^48^ at the same level of theory (Table S1). The energetically attractive formations of the supramolecular complexes Glc-1, 2, 3, 4, and 8 are achieved through the intermolecular non-bonding contacts induced by CH–π, OH–π, and lone pair–π (O:–π) interactions, which have been reported as crucial factors for the formation of supramolecular complexes such as C60-H_2_O^49–51^, CNT-ethylene glycol^52^, and graphene-hexanediol^53^. The conformational flexibility in molecular framework of sugars enables maximal contact on the curved π-surface of fullerene in the C60-Glcs complexes. Indeed, the total number of π-interactions in Glc-1, 2, 3, 4, and 8 linearly increases with the degree of polymerization (DP) in (Glc-*n*) (*n* = 1–4, 8) as 8, 12, 14, 16, and 32 points, respectively and the induced conformational change of Glcs frameworks allows efficient adsorption to the surface of fullerene (Figs. S4–S8, Table S3). It is noteworthy that both the DP and the total number of interaction points in Glc-1, 2, 3, 4, and 8 show proportional relationships to the corresponding *E*_binding_ which clearly illustrate the pivotal role of non-covalent interactions in the excellent fullerene-based separation of Glcs as depicted in Fig. 3b.

As further applications, the retention behaviors toward di-saccharides, including maltose, trehalose, and sucrose without any labeling, were evaluated with the fullerene columns. For the comparison, an original silica-monolith (Silica), amino-monolith (Amino), and ODS-monolith (ODS) were also evaluated. As shown in Scheme S2, Silica ad Amino columns were intermediates of the fullerene columns, therefore, we confirmed the effects of these functional groups for the separation. Furthermore, a PGC column was utilized as a comparison column, which is often used for the separation of saccharides^54–56^. As shown in Table 1 and Fig. S12, the elution order was trehalose, sucrose, and maltose and this order was similar in the PGC column. Although the C70 column also provided the same elution order, the absolute retentions for these analytes were lower. The difference between C60 and C70 may be caused by the immobilization density of fullerenes^37^. Briefly, the density of C60 was almost double that of C70, thus the retentions of small-sized saccharides were not as strong as shown in Fig. 2c,d. Regarding hydrophobicity, the octanol–water partition coefficient (log *P*) of maltose, trehalose, and sucrose are − 5.0, − 3.7, and − 3.3, respectively^57^. Furthermore, the retention differences of these analytes were not observed in the ODS column. According to these considerations, the retention of di-saccharides in the C60 column was not caused by hydrophobicity. Moreover, both the silica and amino columns did not provide any retention of di-saccharides; therefore the functional groups, such as silanol and amino, did not affect the retention of di-saccharides in the C60 column.

**Table 1:** Retention time (RT) of di-saccharides and RDSs.

	**Maltose**		**Sucrose**		**Trehalose**	
	RT (min)	RSD (%)	RT (min)	RSD (%)	RT (min)	RSD (%)
C60^[1]^	15.31	1.36	14.68	0.62	14.19	0.55
C70^[1]^	12.74	1.07	12.47	0.34	12.43	0.54
PGC^[2]^	2.35	0.34	1.90	0.48	1.31	0.65
ODS^[1]^	9.63	0.48	9.61	1.77	9.33	0.48
Silica^[1]^	13.00	0.55	13.09	0.12	12.98	1.89
Amino^[1]^	13.14	0.25	13.29	1.55	13.19	0.25

Conditions: columns, C60 column (75.0 cm × 100 μm i.d.), C70 column (75.0 cm × 100 μm i.d.), PGC column (5.0 cm × 4.6 mm i.d.), ODS column (55.0 cm × 100 μm i.d.), silica column (75.0 cm × 100 μm i.d.), and amino column (75.0 cm × 100 μm i.d.); flow rate, [1] 500 nL min^–1^, [2] 1.0 mL min^–1^; mobile phase, [1] water, [2] 2% 2-propanol aq.; detection, UV 190 nm.

**Figure 4:**
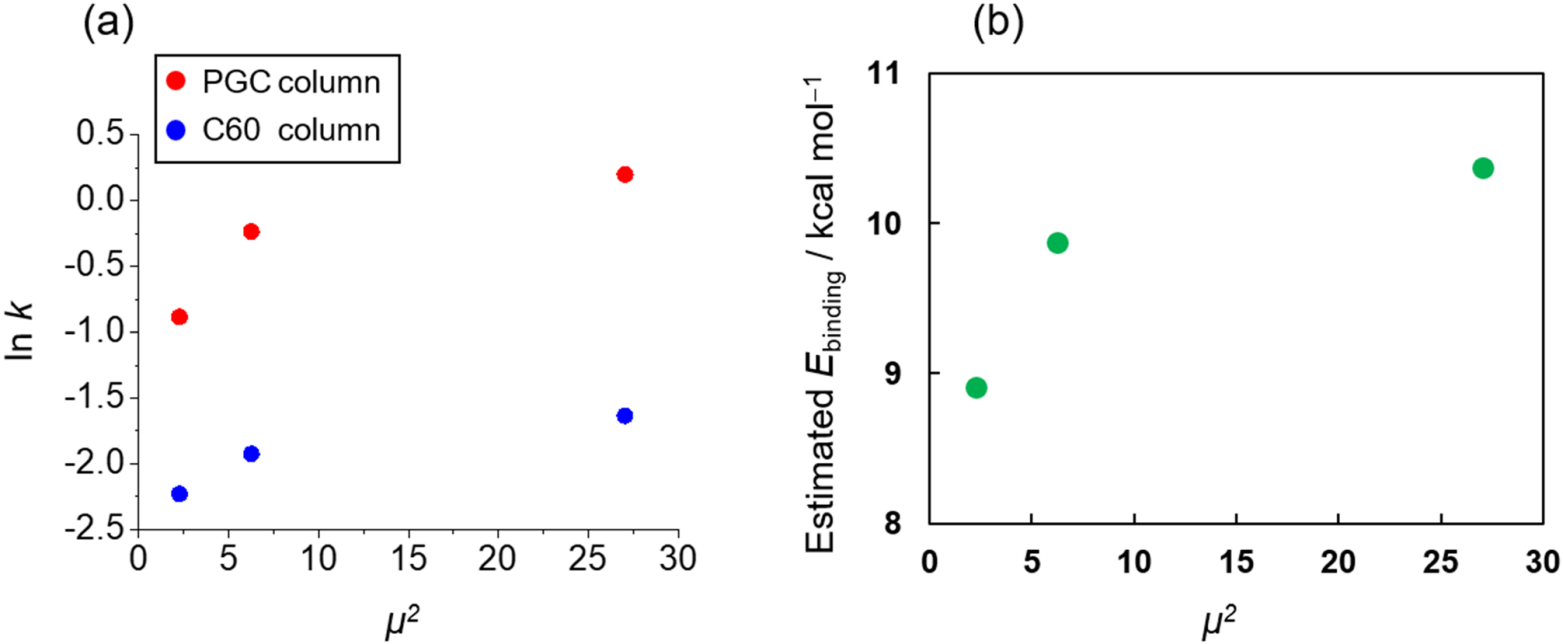
Relationship between the dipole moment and retention of di-saccharides. (**a**) the naturalized logarithm of the retention factor, *k*, (**b**) the estimated binding energy with C60.

As another possibility in the interaction between C60 and di-saccharides, the dipole-induced dipole interaction may potentially work as well as in the PGC column. The dipole moments of trehalose, sucrose, and maltose were previously estimated as 1.5, 2.5, and 5.2, respectively^58^. Figure 4a shows the relation between the square of the dipole moment (*μ*^2^) and the naturalized logarithm of the retention factor (ln *k*) or estimated binding energy. The retention factor, *k*, is defined as follows; *k* = (retention time of the analyte − void time)/void time. According to this figure, the retention behaviors of di-saccharides in the C60 column were similar to the PGC column. These results suggest that the induced dipoles were led by the dipoles of di-saccharides both in the C60 and PGC columns. As shown in Fig. 4b, the theoretical adsorption analysis of C60-disaccharide complexes clearly demonstrates that the order of *E*_binding_ is certainly agreed with the order of retention time and the total number of non-covalent interactions monotonically increases according to the increase of dipole moment of saccharides (Table S2 and Figs. S9–S11).

## Conclusion

In summary, we report the separation of saccharides using fullerene-bonded columns. Both C60 and C70 columns provided effective retention of Glcs through π interactions. Computer simulations also supported the strong interaction between fullerenes and saccharides. Additionally, even in smaller saccharides, the differences in retention due to the dipole moments of di-saccharides were proposed. We believe that in the future, the fullerene-based separation media will be possible to contribute the separation of oligosaccharides and glycoproteins without any labeling in LC.

## Methods

### Chemicals and reagents

Acetone, diethylether, magnesium sulfate, sodium hydroxide, dichloromethane, chlorobenzene, toluene, ethyl acetate, methanol, ethylbenzene, butylbenzene, hexylbenzene, phenyloctane, tetrahydrofuran (THF), chloroform, *n*-hexane were purchased from Nacalai Tesque (Kyoto, Japan), methyl pentafluorobenzoate (MPFB), diethyl amine, 3-aminopropyltrimethoxysilane (APTMS), d-( +)-trehalose dihydrate from Tokyo Chemical Industry (Tokyo, Japan), sodium azide, *N*-hydroxysuccinimide (NHS), 1-ethyl-3-(3-dimethylaminopropyl) carbodiimide (EDAC), d-( +)-glucose, maltose monohydrate, sucrose from Wako Pure Chemical Industries (Osaka, Japan), C_60_-fullerene (C60), C_70_-fullerene (C70), dextran (Mw = 1,000) (glucose homopolymer) from Sigma-Aldrich Japan (Tokyo, Japan), 2-AB Dextran Calibration Ladder (2-AB labeled glucose homopolymer) from Waters (USA), respectively. Deionized water was obtained by a Milli-Q Direct-Q 3UV system (Merck Millipore, Tokyo, Japan). Silica monolithic capillaries (ULTRON HF-SIL, ULTRON HF-ODS) were purchased from Shinwa Chemical Industries Ltd. (Kyoto, Japan). Porous graphite carbon column (PGC column) was purchased from Thermo Fischer Scientific USA).

### Instruments^46^

A capillary liquid chromatographic system consisted of a DiNa S (KYA Technologies, Tokyo, Japan) as a pump, a CE-2070 (JASCO, Tokyo, Japan) as a UV detector, a CHEMINERT (Valco Instruments, Houston, TX) as a sample injector, and a Chemco capillary column conditioner Model 380-b (Chemco, Osaka, Japan) as a column oven. As an HPLC system, a Prominence series (Shimadzu, Kyoto, Japan) was used. FT-IR, NMR, elemental analysis, and fast atom bombardment mass spectrometry (FABMS) were carried out by a Nicolet iS5 ATR (Thermo Fisher Scientific, Yokohama, Japan), a JNM-ECA500 spectrometer (JEOL, Tokyo, Japan), a Flash EA1112 (Thermo Fisher Scientific), and a JMS-700 (JEOL), respectively. LC–MS system was consisted of Ultimate 3,000 nano and Exactive plus (Thermo Fisher Scientific).

### MS conditions

Polarity, negative; spray voltage, 1.8 kV; capillary temp, 295 °C; maximum IT, 150 ms; resolution; 17,500; scan range; 300–2000 m/z.

### Synthesis of 4-azido-2,3,5,6-tetrafluorophenyl succinate

Methyl pentafluorobenzoate (18.3 mmol) and sodium azide (20 mmol) were dissolved in water/acetone = 5/12 (v/v), and the mixture was reacted at 120 °C for 20 h under N_2_ atmosphere. After the extraction with diethyl ether and dehydration with sodium sulfate, the solvents were dried up (PFPA-CO_2_CH_3_). Then, the residue was dissolved in methanol/10% NaOH aq = 4/1 (v/v) and stirred for 30 min. After the neutralization with 6 M HCl and extraction with chloroform, the organic layer was dehydrated and the solvents were removed. The product (PFPA-COOH) (4.09 g, (17.4 mmol) was dissolved in dichloromethane with EDAC (17.2 mmol) and NHS (17.2 mmol), and then the mixture was stirred at room temperature for 16 h under N_2_ atmosphere. The mixture was washed with water to remove unreacted EDAC and NHS. The chloroform layer was dehydrated and the solvent was removed. After silica-gel column chromatography with dichloromethane as a solvent, 4-azido-2,3,5,6-tetrafluorophenyl succinate (NHS-PFPA) was isolated.

### Synthesis of NHS-PFPA conjugated C70 or C60

C70 or C60 (0.12 mmol) and NHS-PFPA (0.24 mmol) were dispersed into chlorobenzene. The mixture was reacted at 108 °C in an oil bath for 120 h under N_2_ atmosphere. After removing the solvent, the residue was purified by silica-gel column chromatography using toluene/ethyl acetate = 10/1 (v/v) as a solvent to remove the excess of unreacted compounds. Then, the products were dissolved in small amount of toluene and add excess of *n*-hexane for precipitation of a product. The precipitation was washed with *n*-hexane and methanol. Finally, the objective products (NHS-PFPA-C70) were isolated and characterizations were carried out by FABMS and elemental analysis.

### Surface modification of the silica monolith with NHS-PFPA-C70 or NHS-PFPA-C60

The procedures were similar to our previous studiesy^36,38,41^. The silica-monolithic capillary was treated with 1.0 M aqueous sodium hydroxide at 40 °C for 3 h. After washing with water and methanol, APTMS in methanol (10%, v/v) was passed through the silica-monolithic capillary for 24 h and washed with methanol. NHS-PFPA-C70 in toluene (8.0 mg mL^−1^) was charged into the NH_2_-monolith for 48 h at room temperature, washed with toluene and methanol.

### Computational details

The initial geometries of 1:1 complex with C_60_ and glucose oligomers (Glcs-*n*) (*n* = 1–4, 8) or disaccharides (trehalose, sucrose, α-maltose) were obtained by simulated annealing (SA) using Forcite program bundled in Material Studio 2019 package. The resulting most stable geometries were further optimized at M06-02X/6-31G(d,p) level of density functional theory (DFT) to evaluate thermodynamic stability of complexation. The SA calculations were performed under a vacuum condition and the subsequent DFT calculation was performed with solvent effect of water based on polarized continuum model PCM).

For SA calculation, the universal force field was used with the cutoff distance 12.5 Å and the parameters of annealing simulation were set as follows: a total of 20 annealing cycles, an initial temperature of 173 K, a midcycle temperature of 2,373 K, 5 heating ramps per cycle, with 200 dynamic steps per ramp. After each cycle, the lowest energy configuration was reoptimized by a molecular dynamics (MD) simulation and van der Waals, electrostatic, and total potential energies of the complexes were calculated based on the results of calculation: simulation length of 5 ps with a 1 fs time step at a temperature of 298 K, taking conformations every 5,000 steps. The force field used was Dreiding 2.21 with Gasteiger charges as implemented in the Materials Studio package. The geometry optimization was performed by using smart algorithm in Forcite module of Materials Studio: convergence tolerance energy of 2 × 10^−5^ kcal/mol, force of 0.001 kcal/mol/Å, and displacement of 1 × 10^−5^ Å with maximum number of iterations of 500 for an independent optimization in the microcanonical ensemble (NVE).

For DFT calculation, the resulting SA-calculated lowest energy geometries of the complexes were further optimized at M06-02X/6-31G(d,p) level calculation including solvent effect of water (PCM) using Gaussian16 package to obtain Gibbs free energy of the complexes with zero-point energy correction. The corresponding biding energy of *E*_binding_ in the C60-sugar complexes were determined by the differences of energy of complex *E*_C60-sugar_ and the sum of energy of isolated C60 (*E*_C60_) and sugar (*E*_*sugar*_) as shown in Eq. (2). The correction for basis set superposition error (BSSE) of

2 $$ E_{{{\text{binding}}}} = E_{{{\text{C6}}0 - {\text{sugar}}}} {-} \, \left( {E_{{{\text{C6}}0}} + E_{{{\text{sugar}}}} } \right) \, + BSSE_{{{\text{correction}}}} $$

*BSSE*_correction_ was calculated at the same level of calculation M06-02X/6-31G(d,p) without solvent effect of water using the Counterpoise key word in Gussian16. Values of *E*_C60-sugar_, *E*_C60_ + *E*_sugar_, *BSSE*_correction_, and *E*_binding_ corresponded to the complexes are summarized in Tables S1–S3. The resulting geometries of of 1:1 complexes are depicted with the numbers of non-bonding interactions of CH–π, OH–π, and O:–π (distances ≤ 3.5 Å) as shown in Figs. S4–S11. Also, the coordinates of the optimized structure of the complexes are summarized in Tables S4–S11.

## Supplementary information

Supplementary Information 1.

Supplementary Information 2.
