# Supplementary material for: Separation of saccharides using fullerene-bonded silica monolithic columns via π interactions in liquid chromatography
Source: Sci Rep. 2020 Aug 14;10:13850. doi: 10.1038/s41598-020-70904-3 (PMC7429847; doi:10.1038/s41598-020-70904-3)
Supplement: Supplementary file 1 — Supplementary Information 1. [file 41598_2020_70904_MOESM1_ESM.pdf]

Supplementary information

Separation of saccharides using fullerene-bonded silica monolithic columns via π interactions in liquid chromatography

Hiroshi KOBAYASHI, Kazuya OKADA, Shinosuke TOKUDA, Eisuke KANAO, Yusuke MASUDA, Toyohiro NAITO, Hikaru TAKAYA, Mingdi YAN, Takuya KUBO,* and Koji OTSUKA

Corresponding author
Takuya Kubo
Tel: +81-75-383-2448
Fax: +81-75-383-2450
E-mail: kubo.takuya.6c@kyoto-u.ac.jp

Contents

Figure S1. Structures of the analytes for the LC evaluations
Scheme S1. Synthesis of NHS-PFPA-C70 and NHS-PFPA-C60
Scheme S2. Preparation of a C70 column and C60 column
Figure S2. FRIR spectra of the synthesized compounds
Figure S3. Separation of 2AB-Glcs with ODS-silica monolithic capillary
Table S1. Values of $E_{\mathrm{C60\text{-}sugar}}$, E_{C60} + E_{sugar}, $BSSE_{\mathrm{correction}}$, and E_{binding} for C60/Glcs
Table S2. Values of $E_{\mathrm{C60\text{-}sugar}}$, E_{C60} + E_{sugar}, $BSSE_{\mathrm{correction}}$, and E_{binding} for C60 and disaccharides
Table S3. Relationships between E_{binding}, the DP of Glc-*n*, and the total number of non-covalent interactions in supramolecular complexes C60/Glcs
Figures S4-11. The lowest energy molecular geometry of the complexes
Tables S4-11. Cartesian coordinates of the optimized structure of the complexes
Figure S12. Chromatograms of di-saccharides

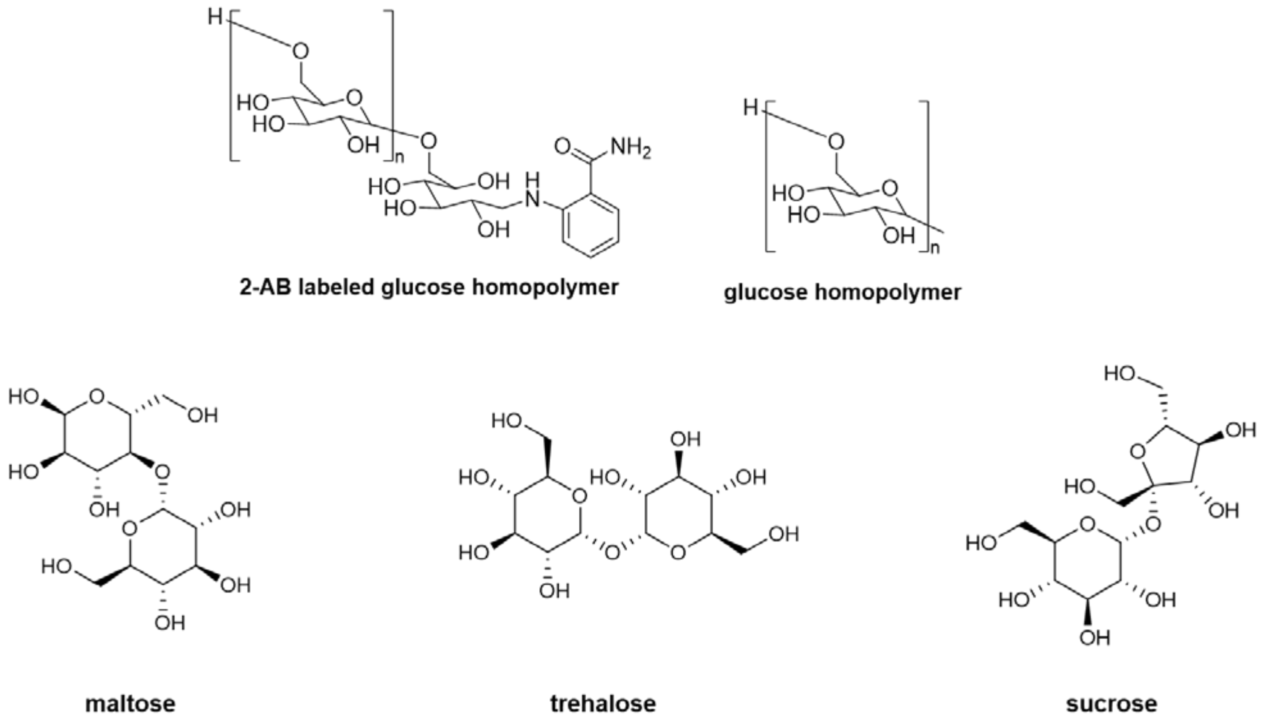

Figure S1. Structures of the analytes for the LC evaluations.

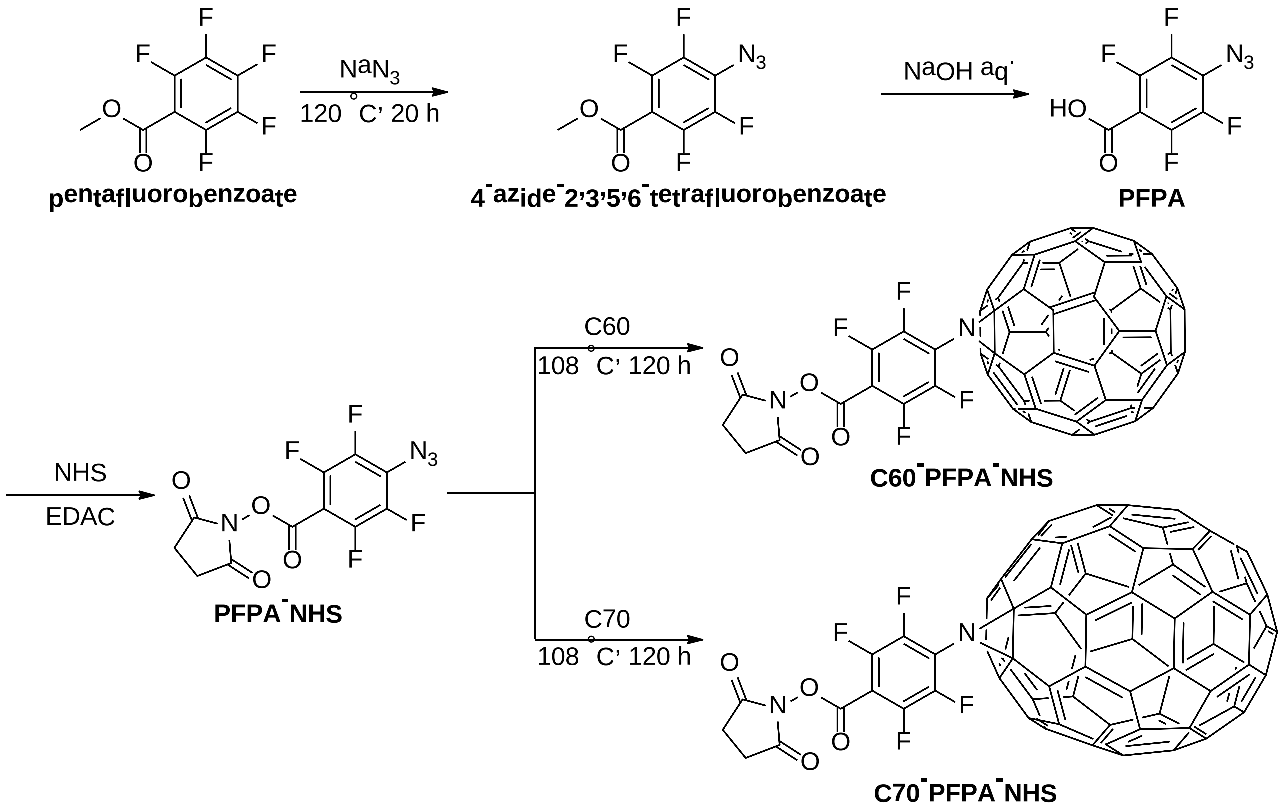

Scheme S1. Synthesis of NHS-PFPA-C70 and NHS-PFPA-C60.

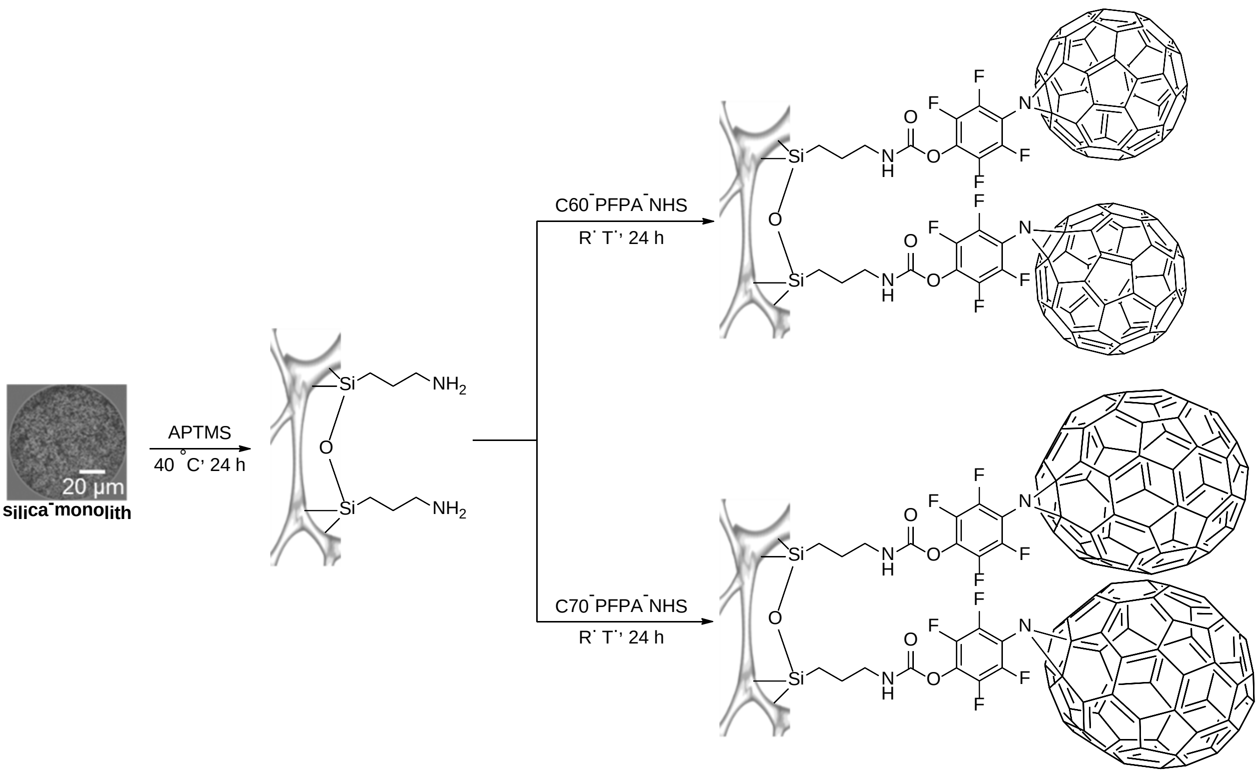

Scheme S2. Preparation of a C70 column and C60 column.

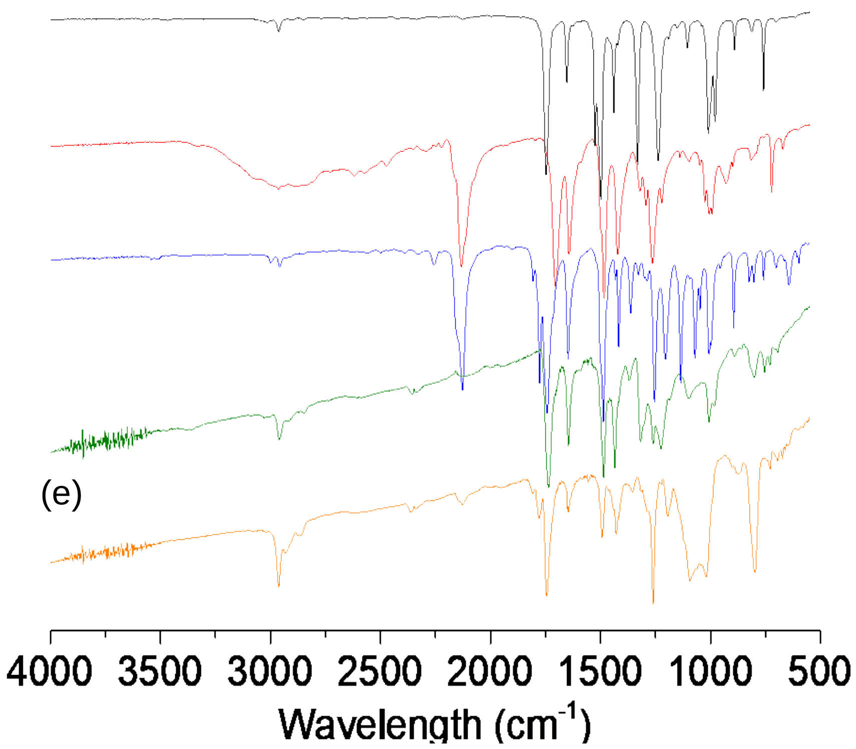

Figure S2. FRIR spectra of the synthesized compounds.
(a) methyl pentafluorobenzoate, (b) PFPA, (c) PFPA-NHS, (d) C60-PFPA-NHS, (e) C70-PFPA-NHS, Peak identities: 1700 cm^{-1} (C=O stretching), 2100 cm^{-1} (azide stretching), 2800 cm^{-1} (O–H stretching, carboxylic acid).

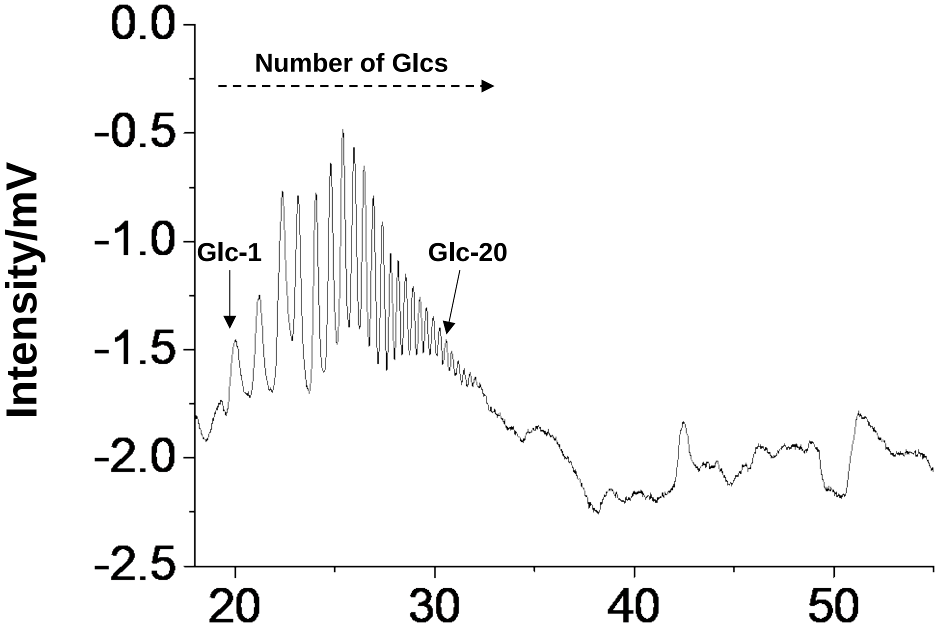

Figure S3. Separation of 2AB-Glcs with ODS-silica monolithic capillary.
LC conditions: columns, ODS silica-monolithic capillary (25.0 cm × 100 μm i.d.), mobile phase, MeCN aq. 2 to 8% MeCN linear gradient for 45 min; detection 214 nm; flow rate, 350 nL/min

Table S1. Values of $E_{C60\text{-sugar}}$, E_{C60} + E_{sugar}, $BSSE_{correction}$, and $E_{binding}$ for **C60/Glcs**.

**Energy (kcal/mol)**	**C_{60}/Glc-1**	**C_{60}/Glc-2**	**C_{60}/Glc-3**	**C_{60}/Glc-4**	**C_{60}/Glc-8**
**$E_{C60\text{-sugar}}$ (BSSE corrected)**	-1865195.53	-2248305.14	-2631423.77	-3014547.34	-4547051.36
**$E_{C60 + Esugar}$**	-1865191.24	-2248298.44	-2631413.75	-3014536.64	-4547029.72
**$BSSE_{correction}$**	3.86	6.52	9.15	9.06	18.19
**$E_{binding}$**	-4.30	-6.70	-10.01	-10.70	-21.64

Table S2. Values of $E_{C60\text{-sugar}}$, E_{C60} + E_{sugar}, $BSSE_{correction}$, and $E_{binding}$ for C60 and disaccharides.

**Energy (kcal/mol)**	**$C_{60/}$trehalose**	**C_{60}/sucrose**	**$C_{60/}\alpha$-maltose**
**$E_{C60\text{-sugar}}$ (BSSE corrected)**	-2248307.12	-2248309.77	-2248309.91
**$E_{C60 + Esugar}$**	-2248301.51	-2248303.06	-2248302.27
**$BSSE_{correction}$**	5.50	5.50	5.99
**$E_{binding}$**	-5.61	-6.70	-7.63

Table S3. Relationships between $E_{binding}$, the DP of Glc-*n*, and the total number of non-covalent interactions in supramolecular complexes **C60/Glc-1,2,3,4,8**.

**$E_{binding}$ (kcal/mol)**	***n* of $(Glcs)_n$**	**No. of interactions**
-4.3	1	8
-6.7	2	12
-10.01	3	14
-10.7	4	16
-21.64	8	32

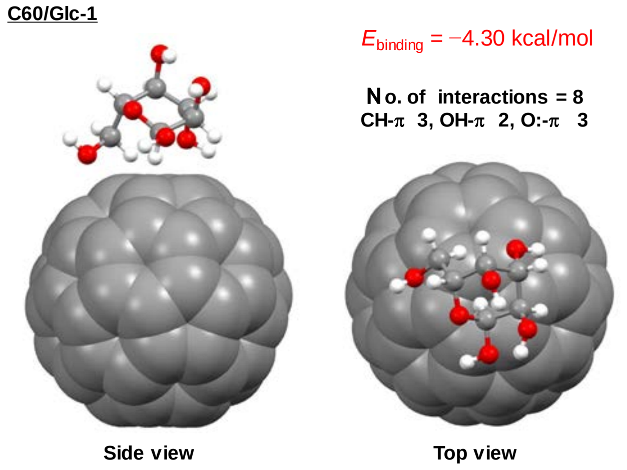

Figure S4. The lowest energy molecular geometry of C60/Glc-1.

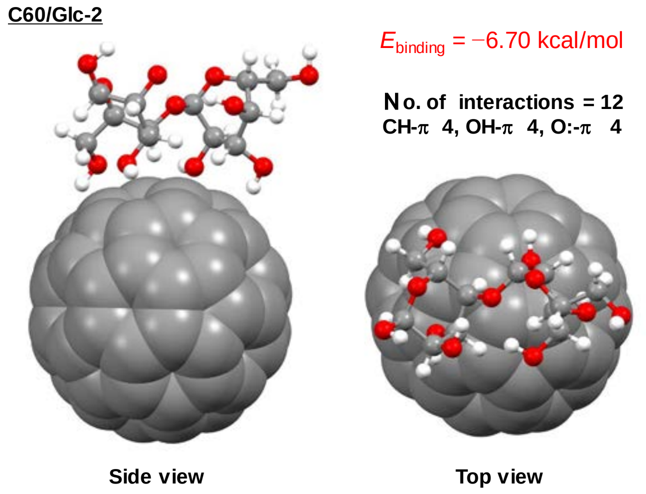

Figure S5. The lowest energy molecular geometry of C60/Glc-2.

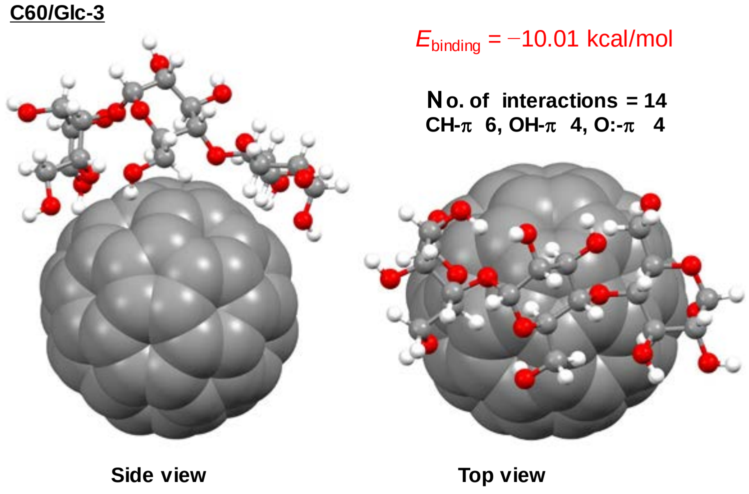

Figure S6. The lowest energy molecular geometry of C60/Glc-3.

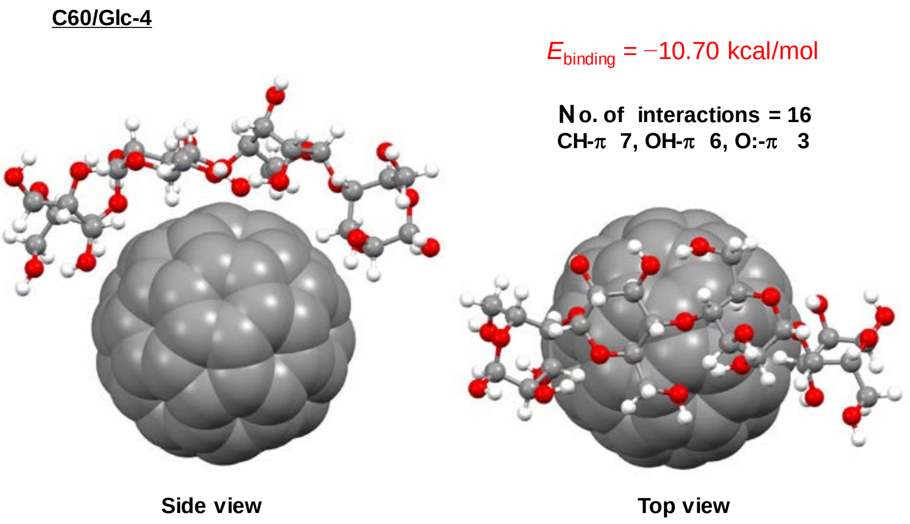

Figure S7. The lowest energy molecular geometry of C60/Glc-4.

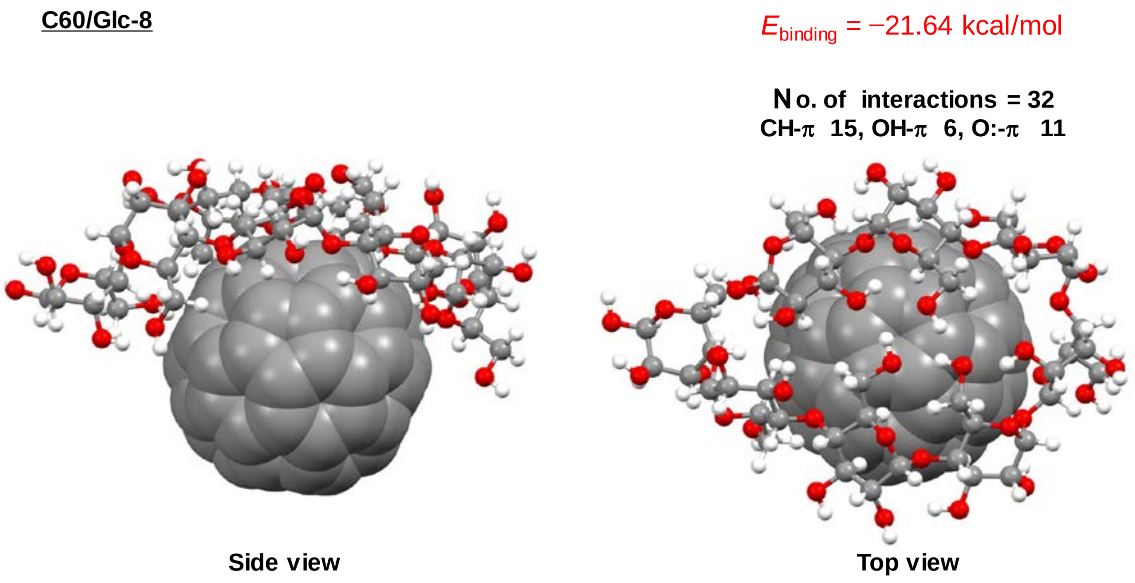

Figure S8. The lowest energy molecular geometry of C60/Glc-8.

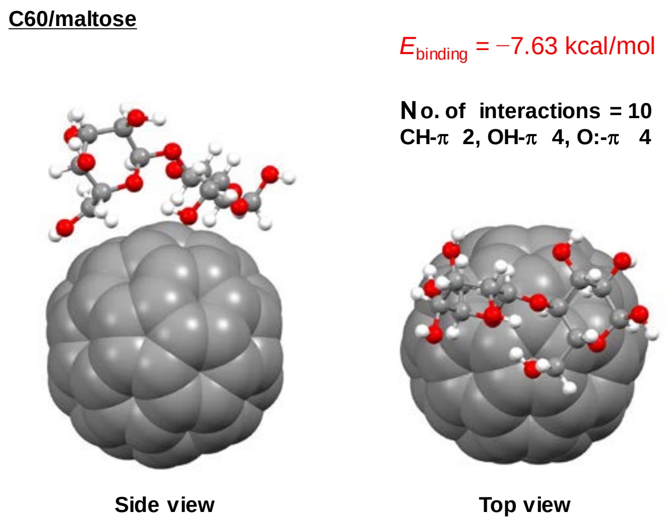

Figure S9. The lowest energy molecular geometry of C60/maltose.

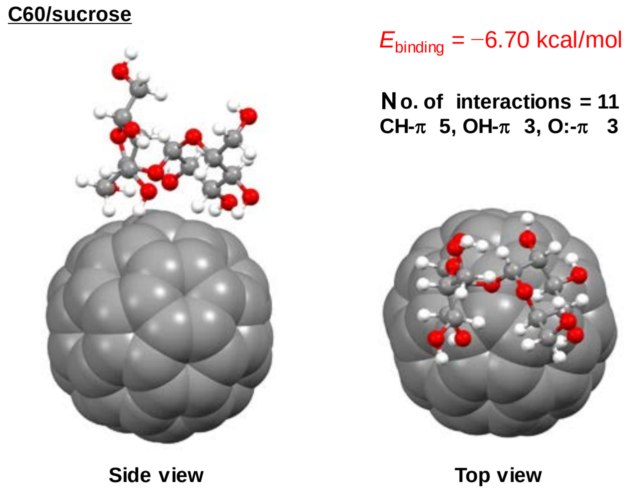

Figure S10. The lowest energy molecular geometry of C60/sucrose.

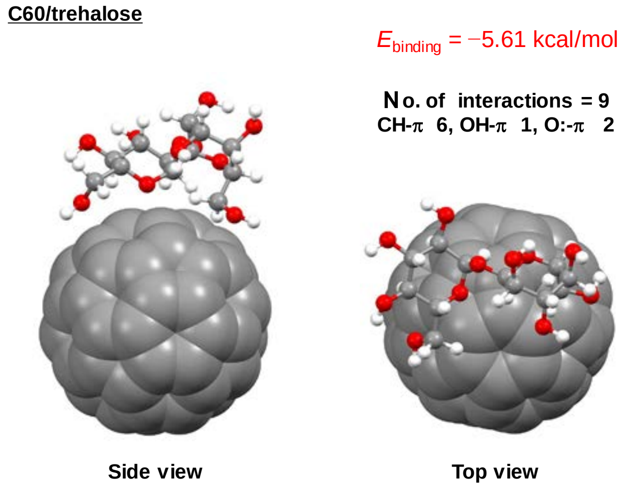

Figure S11. The lowest energy molecular geometry of C60/trehalose.

Table S4. Cartesian coordinates of the optimized structure of C_{60}/Glc-1.

No.	Symbol	X	Y	Z	No.	Symbol	X	Y	Z
1	C	4.87857	-1.150547	-0.131263	36	C	3.542606	-2.788255	0.89207
2	C	4.889144	-0.151237	-1.09348	37	C	2.276154	-3.354534	0.908093
3	C	4.741903	-0.805465	1.27176	38	C	3.916357	-1.81777	1.904154
4	C	4.15919	-0.331334	-2.334856	39	C	-0.896326	-1.944481	1.720894
5	C	4.137223	-2.375922	-0.365933	40	C	-0.521999	-0.97316	2.732877
6	C	4.633026	1.569059	0.644149	41	C	1.378802	2.023813	-2.878239
7	C	4.763738	1.239234	-0.697089	42	C	1.735673	2.951565	-1.910148
8	C	4.622075	0.523763	1.650837	43	C	0.054052	1.431379	-2.860975
9	C	3.450584	-1.502758	-2.559282	44	C	3.053464	2.897795	-1.304634
10	C	3.439356	-2.548076	-1.552558	45	C	2.32296	0.999429	-3.284603
11	C	3.094513	2.181502	2.309026	46	C	-0.482378	2.76477	-0.865948
12	C	1.754018	2.436985	2.559192	47	C	0.784286	3.330388	-0.881553
13	C	3.671062	0.90206	2.679826	48	C	-0.856202	1.794565	-1.87881
14	C	0.946489	3.116615	1.563131	49	C	3.956175	1.918482	-1.693401
15	C	3.689078	2.593629	1.050957	50	C	3.582656	0.947919	-2.705611
16	C	1.479222	0.201345	3.545913	51	C	-1.809852	1.124558	0.15844
17	C	0.928219	1.424474	3.191549	52	C	-1.825425	0.126664	1.120739
18	C	2.881528	-0.065625	3.284024	53	C	-1.67753	0.780589	-1.245262
19	C	1.514659	3.510296	0.360143	54	C	-1.097995	0.306421	2.362773
20	C	2.916735	3.242718	0.098454	55	C	-1.076056	2.352251	0.392802
21	C	-0.03342	-2.206311	-2.282545	56	C	-1.572479	-1.594849	-0.617585
22	C	1.307216	-2.461307	-2.532455	57	C	-1.70463	-1.26601	0.724359
23	C	-0.609572	-0.927141	-2.654021	58	C	-1.561041	-0.548471	-1.625507
24	C	2.114619	-3.140453	-1.535782	59	C	-0.389412	1.478422	2.586409
25	C	-0.628571	-2.61859	-1.024326	60	C	-0.378188	2.52381	1.579592
26	C	1.581656	-0.226182	-3.519464	61	O	-5.830534	0.352737	-1.294174
27	C	2.132849	-1.449166	-3.165032	62	O	-8.153034	0.099315	0.474811
28	C	0.17945	0.040823	-3.258428	63	H	-6.698109	-1.231906	2.037138
29	C	1.546096	-3.534652	-0.333208	64	C	-4.923014	-0.629258	-0.844004
30	C	0.14369	-3.267573	-0.071334	65	C	-6.878452	0.558524	0.879158
31	C	1.682266	-2.048522	2.904503	66	C	-6.191386	1.290884	-0.282599
32	C	1.324992	-2.976532	1.936922	67	H	-7.028417	1.255099	1.711549
33	C	3.006886	-1.456027	2.887515	68	O	-4.934423	-0.121521	2.104218
34	C	0.006809	-2.923506	1.331684	69	O	-4.239507	2.594052	-0.96576
35	C	0.738026	-1.024268	3.311414	70	H	-4.021235	-0.148456	-0.443697

(continued).

No.	Symbol	X	Y	Z
71	H	-4.827341	-2.230213	0.579458
72	H	-4.256619	-0.921704	-2.628111
73	H	-4.479554	-0.856775	2.534399
74	H	-6.952252	1.91912	-0.759617
75	C	-5.015421	2.180897	0.140476
76	O	-4.635139	-1.46214	-1.921329
77	O	-6.703012	-2.156969	-0.324252
78	C	-5.571541	-1.5018	0.222446
79	C	-6.045629	-0.630109	1.391402
80	H	-7.988205	-0.641183	-0.131496
81	H	-5.427983	3.035652	0.695669
82	H	-6.458678	-2.424128	-1.223382
83	H	-4.33622	1.651621	0.810408
84	H	-4.829521	2.994658	-1.616923

Table S5. Cartesian coordinates of the optimized structure of C_{60}/Glc-2.

No.	Symbol	X	Y	Z
1	C	5.613386	0.467929	-1.685176
2	C	5.073893	1.742351	-1.783054
3	C	6.061323	-0.033645	-0.399059
4	C	3.904246	1.973742	-2.610477
5	C	5.007817	-0.634000	-2.410042
6	C	5.385855	2.094635	0.630018
7	C	4.957796	2.574268	-0.599466
8	C	5.950126	0.761417	0.732418
9	C	3.325352	0.920360	-3.303488
10	C	3.889659	-0.412826	-3.201006
11	C	4.665877	1.155890	2.658459
12	C	3.549126	0.738028	3.367901
13	C	5.505036	0.181295	1.986080
14	C	2.307380	1.483134	3.271835
15	C	4.592282	2.338446	1.820427
16	C	4.022995	-1.605576	2.795625
17	C	3.220463	-0.673704	3.437862
18	C	5.190615	-1.168294	2.052900
19	C	2.237059	2.613633	2.471039
20	C	3.405208	3.050845	1.728895
21	C	0.434359	-1.035633	-2.576230
22	C	1.551715	-0.618622	-3.285801
23	C	-0.404586	-0.061861	-1.902876
24	C	2.793344	-1.363894	-3.189925
25	C	0.508020	-2.219605	-1.737619
26	C	1.078549	1.723940	-2.711941
27	C	1.880738	0.792894	-3.355946
28	C	-0.090052	1.287535	-1.971167
29	C	2.863958	-2.494662	-2.389140
30	C	1.696164	-2.931676	-1.646556
31	C	4.211165	-2.951319	0.880052
32	C	3.588771	-3.306040	-0.308184
33	C	5.307171	-2.000161	0.869281
34	C	2.144110	-3.433160	-0.360927
35	C	3.417347	-2.707295	2.070541
36	C	5.081677	-1.816731	-1.571926
37	C	4.033879	-2.726140	-1.561880
38	C	5.732757	-1.445646	-0.329168
39	C	1.385403	-3.198102	0.776781
40	C	2.036393	-2.828424	2.020129
41	C	0.890260	3.069846	-0.797454
42	C	1.512575	3.425097	0.390574
43	C	-0.207695	2.120697	-0.788083
44	C	2.957478	3.552341	0.442896
45	C	1.684020	2.826472	-1.988046
46	C	0.018453	1.936225	1.654002
47	C	1.067185	2.845010	1.643619
48	C	-0.634871	1.565605	0.411618
49	C	3.716149	3.319426	-0.694983
50	C	3.064975	2.948290	-1.937999
51	C	-0.515071	-0.348705	1.767416
52	C	0.025699	-1.624067	1.866112
53	C	-0.965817	0.152451	0.481608
54	C	1.196817	-1.853481	2.691747
55	C	0.092568	0.752932	2.491429
56	C	-0.286584	-1.975357	-0.547126
57	C	0.143305	-2.455081	0.681556
58	C	-0.847279	-0.642142	-0.649268
59	C	1.775585	-0.800825	3.385599
60	C	1.211142	0.531963	3.282319
61	O	-6.703706	1.462739	-1.001065
62	C	-5.512820	0.798598	-1.273508
63	C	-5.486843	2.357662	1.005570
64	C	-6.691396	2.437970	0.060370
65	H	-5.451594	3.317341	1.530777
66	O	-5.655054	1.373627	2.006571
67	H	-3.575931	1.414308	0.740871
68	O	-3.442949	3.436712	0.302051
69	H	-4.660153	2.664235	-1.798164
70	O	-5.402950	-0.229389	-0.293359

(continued).

No.	Symbol	X	Y	Z
71	O	-3.115988	1.303289	-1.674614
72	H	-7.588302	2.238668	0.659237
73	C	-6.827292	3.838915	-0.547389
74	H	-5.609498	0.355988	-2.272785
75	C	-4.354248	1.792129	-1.206670
76	C	-4.148634	2.212556	0.243491
77	O	-7.167073	4.787956	0.442201
78	H	-5.821638	0.526565	1.566223
79	H	-2.626949	3.307650	-0.203944
80	H	-5.873436	4.171421	-0.968027
81	H	-7.564889	3.799076	-1.357959
82	H	-8.069762	4.608917	0.733436
83	O	-5.757845	-3.111484	-0.725845
84	C	-5.484840	-3.646865	0.567534
85	C	-4.320820	-1.168631	-0.379311
86	C	-4.720019	-2.325136	-1.298132
87	H	-3.414077	-0.694693	-0.759817
88	H	-4.026482	-0.721802	1.700397
89	O	-2.908196	-2.376945	1.105488
90	H	-5.054469	-2.890918	2.536368
91	O	-6.577797	-4.408672	0.938609
92	O	-6.486907	-1.760729	1.653715
93	H	-5.159496	-1.897589	-2.206747
94	C	-3.527488	-3.167054	-1.756429
95	H	-4.607389	-4.302347	0.536737
96	C	-5.273195	-2.487481	1.542454
97	C	-4.100411	-1.619100	1.069812
98	O	-2.758442	-2.323662	-2.599696
99	H	-2.632044	-2.480199	2.025090
100	H	-7.257312	-3.778559	1.226204
101	H	-6.677394	-1.405181	0.769446
102	H	-2.933773	-3.515878	-0.905054
103	H	-3.920198	-4.036301	-2.300491
104	H	-1.935778	-2.779974	-2.812634
105	H	-3.127778	1.250235	-2.638703

Table S6. Cartesian coordinates of the optimized structure of C_{60}/Glc-3.

No.	Symbol	X	Y	Z
1	C	-1.572869	-2.967532	1.958018
2	C	-2.710762	-3.513283	1.380883
3	C	-0.412275	-2.673113	1.138445
4	C	-4.023199	-3.035642	1.7747
5	C	-1.693911	-1.921853	2.956726
6	C	-1.630501	-3.502671	-0.82735
7	C	-2.740478	-3.784909	-0.043572
8	C	-0.440957	-2.935028	-0.223114
9	C	-4.139501	-2.034985	2.728727
10	C	-2.948751	-1.465931	3.333176
11	C	-0.718927	-1.95318	-2.337904
12	C	-0.956846	-0.757545	-2.99998
13	C	0.120414	-1.97598	-1.155468
14	C	-2.287426	-0.447501	-3.489246
15	C	-1.802142	-2.89786	-2.136386
16	C	0.437002	0.448706	-1.375368
17	C	-0.362888	0.472729	-2.509694
18	C	0.693044	-0.805005	-0.686088
19	C	-3.323146	-1.349301	-3.293008
20	C	-3.075013	-2.601584	-2.601781
21	C	-4.745137	1.4607	2.375588
22	C	-4.508375	0.263581	3.035611
23	C	-5.587229	1.483261	1.193677
24	C	-3.177208	-0.045499	3.523385
25	C	-3.661204	2.403782	2.172595
26	C	-5.908348	-0.944164	1.415132
27	C	-5.103286	-0.965924	2.544837
28	C	-6.155488	0.308013	0.724264
29	C	-2.140432	0.856135	3.329932
30	C	-2.388071	2.108545	2.639979
31	C	0.492646	0.88736	0.928078
32	C	-0.261321	1.334525	2.005934
33	C	0.729261	-0.530726	0.741423
34	C	-1.228669	2.405929	1.819785
35	C	0.315401	1.49251	-0.378599
36	C	-0.60805	-0.980383	2.75383
37	C	-0.827238	0.378178	2.938426
38	C	0.187127	-1.44421	1.633228
39	C	-1.393599	2.983027	0.568365
40	C	-0.603872	2.514723	-0.555752
41	C	-5.957389	-1.385099	-0.890714
42	C	-5.199582	-1.828466	-1.965122
43	C	-6.18586	0.03542	-0.700876
44	C	-4.235758	-2.897585	-1.781301
45	C	-5.786457	-1.990521	0.417043
46	C	-4.854201	0.486646	-2.7176
47	C	-4.635606	-0.871554	-2.899139
48	C	-5.646797	0.95023	-1.593682
49	C	-4.072389	-3.476299	-0.531298
50	C	-4.864855	-3.012728	0.592605
51	C	-3.892633	2.476191	-1.923205
52	C	-2.754789	3.020155	-1.345073
53	C	-5.05256	2.179892	-1.102454
54	C	-1.442677	2.542113	-1.739066
55	C	-3.770331	1.429679	-2.921302
56	C	-3.832927	3.009092	0.865015
57	C	-2.72431	3.293284	0.080626
58	C	-5.023679	2.44047	0.259811
59	C	-1.325676	1.542559	-2.694475
60	C	-2.51557	0.973199	-3.299048
61	O	2.272814	5.323684	1.416391
62	C	1.801441	5.997593	0.26864
63	C	3.292986	3.532058	0.144872
64	C	2.323752	3.906536	1.269346
65	H	4.30943	3.832702	0.434199
66	O	3.23048	2.127414	-0.043735
67	H	1.912234	3.858475	-1.451106
68	O	3.873383	3.991579	-2.123961
69	H	3.750917	6.126262	-0.587244
70	O	0.530875	5.563739	-0.120906

(continued).

No.	Symbol	X	Y	Z
71	O	2.348524	6.315116	-2.110832
72	H	1.324328	3.538726	1.002633
73	C	2.728921	3.313484	2.606433
74	H	1.793054	7.058407	0.546609
75	C	2.766867	5.742319	-0.889631
76	C	2.894424	4.246205	-1.13664
77	O	1.761517	3.557927	3.609391
78	H	3.647419	4.55702	-2.876185
79	H	2.919075	2.24213	2.468087
80	H	3.661389	3.779688	2.938637
81	H	0.983852	3.024572	3.400117
82	H	2.56586	7.254674	-2.108067
83	O	5.285748	-0.385117	-1.682897
84	C	5.93023	-0.976314	-0.585751
85	C	4.446888	1.501247	-0.411026
86	C	4.104194	0.344259	-1.347753
87	H	5.109637	2.210245	-0.924048
88	H	4.391891	0.307718	1.350847
89	O	5.535767	2.013905	1.650758
90	H	7.078726	0.733048	-0.01565
91	O	5.106508	-1.905627	0.056442
92	O	6.798471	-0.484448	1.649876
93	H	3.399938	-0.314566	-0.814902
94	C	3.471906	0.784026	-2.654451
95	H	6.816407	-1.474708	-0.998588
96	C	6.324071	0.082926	0.447044
97	C	5.127228	0.942425	0.828
98	O	2.969389	-0.323909	-3.382059
99	H	6.017522	1.621494	2.3924
100	H	2.694721	1.527069	-2.443508
101	H	4.235113	1.259714	-3.277285
102	H	2.145172	-0.60306	-2.962834
103	O	2.656483	-3.221482	0.237305
104	C	2.959357	-4.291418	1.123898
105	C	4.898032	-3.143906	-0.624263
106	C	3.41019	-3.231347	-0.969326
107	H	5.505698	-3.1897	-1.535726
108	H	6.361478	-4.014403	0.682785
109	O	5.267581	-5.460364	-0.352852
110	H	4.605482	-5.161302	2.161333
111	O	2.136342	-4.167796	2.232769
112	O	4.683263	-3.161031	2.446528
113	H	3.142109	-2.302063	-1.484819
114	C	3.034926	-4.360856	-1.92301
115	H	2.790342	-5.247886	0.606271
116	C	4.417518	-4.244371	1.587785
117	C	5.337687	-4.238638	0.352324
118	O	1.697502	-4.126028	-2.32602
119	H	5.602789	-6.16444	0.216243
120	H	1.227465	-4.309306	1.936053
121	H	4.858896	-2.383141	1.894506
122	H	3.725399	-4.319574	-2.778078
123	H	3.150489	-5.34362	-1.458478
124	H	1.414997	-4.868142	-2.872757
125	H	7.70085	-0.800751	1.519785
126	H	-0.08593	5.759423	0.597175

Table S7. Cartesian coordinates of the optimized structure of C_{60}/Glc-4.

No.	Symbol	X	Y	Z
1	C	-2.666145	3.001533	2.408503
2	C	-2.790019	1.704962	1.929574
3	C	-1.525419	3.365859	3.22817
4	C	-3.22999	1.474216	0.565535
5	C	-2.974854	4.125217	1.543865
6	C	-0.690273	1.060888	3.035182
7	C	-1.781066	0.712392	2.251616
8	C	-0.559545	2.417597	3.533433
9	C	-3.529019	2.547855	-0.261102
10	C	-3.395289	3.903318	0.240099
11	C	1.584966	1.641481	2.973235
12	C	2.671269	1.846901	2.1342
13	C	0.846556	2.776001	3.495796
14	C	2.854883	1.002747	0.968484
15	C	0.635781	0.580781	2.689175
16	C	2.359286	4.281755	2.275469
17	C	3.065442	3.196916	1.776247
18	C	1.224974	4.0665	3.15459
19	C	1.944782	-0.007193	0.694406
20	C	0.812014	-0.225422	1.574922
21	C	-1.617358	4.094399	-2.844238
22	C	-2.701436	3.887252	-2.003056
23	C	-0.878921	2.960183	-3.367544
24	C	-2.88541	4.731811	-0.836997
25	C	-0.667858	5.153891	-2.557352
26	C	-2.393699	1.453337	-2.147778
27	C	-3.100093	2.537573	-1.647839
28	C	-1.257499	1.669802	-3.025001
29	C	-1.978342	5.745123	-0.563218
30	C	-0.844215	5.96073	-1.442681
31	C	0.724394	5.88641	1.757959
32	C	-0.132467	6.335187	0.763342
33	C	0.214769	5.0582	2.835179
34	C	0.296635	6.325232	-0.622971
35	C	2.049634	5.406379	1.411696
36	C	-2.02549	5.185186	1.830245
37	C	-1.538373	5.976937	0.800418
38	C	-1.129273	4.715621	2.870703
39	C	1.563695	5.866871	-0.953705
40	C	2.459927	5.397129	0.086615
41	C	-0.754946	-0.146734	-1.627107
42	C	0.100531	-0.594844	-0.631602
43	C	-0.246953	0.678065	-2.706772
44	C	-0.327829	-0.587967	0.753745
45	C	-2.081014	0.330825	-1.283058
46	C	1.993299	0.54908	-1.702974
47	C	1.503161	-0.235516	-0.66911
48	C	1.097443	1.020404	-2.742141
49	C	-1.595616	-0.131534	1.084503
50	C	-2.488271	0.341528	0.043303
51	C	2.634177	2.734906	-2.279338
52	C	2.758526	4.031017	-1.799919
53	C	1.49329	2.370105	-3.098762
54	C	3.198132	4.261869	-0.436179
55	C	2.944743	1.609212	-1.416248
56	C	0.657608	4.67412	-2.903229
57	C	1.748203	5.022709	-2.119683
58	C	0.526976	3.318192	-3.403872
59	C	3.495589	3.187223	0.389605
60	C	3.365195	1.830706	-0.11017
61	O	-3.276224	-4.002854	-0.797605
62	C	-4.058568	-3.788781	0.36353
63	C	-1.189978	-4.27444	0.417201
64	C	-1.915391	-3.559264	-0.731022
65	H	-1.111839	-5.346994	0.179132
66	O	0.088959	-3.690058	0.569707
67	H	-1.966349	-3.023224	1.953822
68	O	-1.381324	-4.850981	2.742509
69	H	-3.464997	-5.612031	1.323209
70	O	-4.088911	-2.441053	0.734024

(continued 1).

No.	Symbol	X	Y	Z
71	O	-4.152994	-4.329873	2.729009
72	H	-1.885475	-2.477186	-0.550468
73	C	-1.378382	-3.875667	-2.120713
74	H	-5.057588	-4.161985	0.114732
75	C	-3.435117	-4.538224	1.533015
76	C	-1.986957	-4.096128	1.703425
77	O	-0.082475	-3.333677	-2.286909
78	H	-2.031315	-4.896315	3.461575
79	H	-1.379009	-4.965465	-2.259864
80	H	-2.073974	-3.437687	-2.847604
81	H	0.170789	-3.434251	-3.212881
82	O	-7.157812	-2.96035	-0.198147
83	C	-7.320591	-2.472232	-1.529065
84	C	-5.129439	-1.606933	0.218091
85	C	-6.515338	-2.076604	0.706994
86	H	-4.915877	-0.63813	0.677555
87	H	-3.989082	-1.567629	-1.602348
88	O	-5.279056	-0.020244	-1.613853
89	H	-6.075305	-1.756698	-3.126319
90	O	-8.04429	-3.42407	-2.22501
91	O	-5.440226	-3.576279	-2.397176
92	H	-6.348477	-2.676907	1.609255
93	C	-7.404431	-0.912746	1.127397
94	H	-7.898377	-1.542746	-1.522739
95	C	-5.944525	-2.273923	-2.16722
96	C	-5.029911	-1.376011	-1.31224
97	O	-7.435289	0.068396	0.096123
98	H	-6.062462	0.252533	-1.103412
99	H	-7.418966	-4.14639	-2.396797
100	H	-4.527868	-3.662477	-2.078406
101	H	-8.410726	-1.298582	1.324269
102	H	-7.001546	-0.483553	2.052382
103	H	-7.933601	0.83717	0.3988
104	H	-4.223862	-3.369483	2.842944
105	O	3.440069	-4.350719	1.602041

No.	Symbol	X	Y	Z
106	C	3.97751	-4.215376	0.32463
107	C	1.128786	-4.578831	0.966132
108	C	2.156698	-3.750361	1.754343
109	H	0.728442	-5.387155	1.591746
110	H	1.003921	-5.295015	-1.05482
111	O	2.274834	-6.487258	0.084668
112	H	3.358553	-4.763146	-1.676919
113	O	4.646209	-2.969653	0.311564
114	O	2.419437	-3.015378	-1.077449
115	H	2.156598	-2.733243	1.344406
116	C	1.891703	-3.705473	3.25321
117	H	4.696043	-5.036124	0.206869
118	C	2.900399	-4.306379	-0.785617
119	C	1.775882	-5.214965	-0.275978
120	O	0.656685	-3.107518	3.562672
121	H	2.56023	-6.939538	-0.719121
122	H	1.967549	-4.729115	3.65109
123	H	2.680471	-3.109184	3.722754
124	H	-0.045773	-3.738435	3.326407
125	O	7.481302	-2.297068	0.102241
126	C	7.444586	-0.87819	-0.017624
127	C	5.278541	-2.576553	-0.904819
128	C	6.743867	-3.017405	-0.874972
129	H	4.781936	-3.007745	-1.778472
130	H	4.056974	-0.860017	-0.723038
131	O	5.462487	-0.611391	-2.243945
132	H	5.988073	0.698908	-0.048883
133	O	8.213075	-0.361578	1.011203
134	O	5.552453	-0.619379	1.428053
135	H	6.769409	-4.061222	-0.539803
136	C	7.417252	-2.980743	-2.245772
137	H	7.890079	-0.559194	-0.967814
138	C	5.999528	-0.38761	0.10474
139	C	5.109974	-1.057282	-0.947868
140	O	6.75387	-3.953525	-3.034836

(continued 2).

No.	Symbol	X	Y	Z
141	H	5.212425	0.319584	-2.315635
142	H	7.650927	-0.424236	1.800827
143	H	5.278314	-1.550279	1.477779
144	H	7.338799	-1.985723	-2.695197
145	H	8.479572	-3.222918	-2.106376
146	H	7.041157	-3.848538	-3.948495
147	H	1.474142	-3.072365	-1.310964

Table S8. Cartesian coordinates of the optimized structure of C_{60}/Glc-8.

No.	Symbol	X	Y	Z
1	C	0.930621	-2.922531	0.723046
2	C	1.430135	-1.768986	0.140217
3	C	1.447473	-3.367247	2.005369
4	C	0.53358	-0.832078	-0.504269
5	C	-0.495093	-3.186639	0.697148
6	C	2.970053	-1.43755	2.023865
7	C	2.478223	-1.013691	0.797973
8	C	2.447616	-2.644612	2.640899
9	C	-0.830552	-1.083772	-0.530643
10	C	-1.356875	-2.286094	0.086029
11	C	2.864598	-1.078043	4.341424
12	C	2.266107	-0.308183	5.328073
13	C	2.37892	-2.419679	4.07269
14	C	2.010271	1.101844	5.097607
15	C	3.230752	-0.472325	3.074519
16	C	0.693614	-2.12676	5.839286
17	C	1.158052	-0.845193	6.096599
18	C	1.317178	-2.931721	4.804896
19	C	2.361896	1.683734	3.88808
20	C	2.982816	0.876715	2.854492
21	C	-3.470456	0.220728	1.463978
22	C	-2.886945	-0.547174	0.467155
23	C	-2.977295	1.556217	1.73618
24	C	-2.627971	-1.95452	0.703744
25	C	-3.843245	-0.383942	2.729078
26	C	-1.306573	1.27086	-0.030802
27	C	-1.776726	-0.008837	-0.296465
28	C	-1.924777	2.075338	1.001515
29	C	-2.980537	-2.537177	1.912908
30	C	-3.601916	-1.732638	2.949322
31	C	-0.992749	-3.35651	4.762445
32	C	-2.1399	-3.249161	3.989219
33	C	0.274884	-3.691135	4.139598
34	C	-3.082193	-2.172464	4.231863
35	C	-0.73332	-2.390275	5.813529
36	C	-0.86254	-3.794934	1.961263
37	C	-2.074774	-3.471849	2.555985
38	C	0.338111	-3.905592	2.770392
39	C	-2.833716	-1.249067	5.237606
40	C	-1.632862	-1.359925	6.044995
41	C	0.3782	2.503029	1.042424
42	C	1.530714	2.402801	1.813725
43	C	-0.88861	2.83979	1.665269
44	C	2.468512	1.320211	1.568988
45	C	0.119121	1.532788	-0.007225
46	C	0.249431	2.93991	3.841307
47	C	1.463948	2.625179	3.246968
48	C	-0.951118	3.054842	3.03453
49	C	2.223658	0.395209	0.563462
50	C	1.020517	0.506817	-0.245784
51	C	-1.544483	2.072616	5.082975
52	C	-2.047286	0.92129	5.671965
53	C	-2.06043	2.519249	3.801898
54	C	-1.146868	-0.018888	6.313536
55	C	-0.116917	2.334523	5.108416
56	C	-3.580038	0.582884	3.780629
57	C	-3.089623	0.160515	5.007476
58	C	-3.052486	1.787414	3.165072
59	C	0.217121	0.232419	6.339374
60	C	0.743664	1.435696	5.72195
61	O	-0.650853	4.139551	-3.478171
62	C	0.203847	4.912999	-2.667902
63	C	-2.556134	4.423992	-1.967056
64	C	-1.66617	3.443297	-2.741148
65	H	-3.151293	5.025135	-2.666971
66	O	-3.389942	3.656997	-1.116232
67	H	-1.195214	4.703266	-0.351027
68	O	-2.49629	6.30868	-0.493893
69	H	-1.067033	6.634238	-2.712809
70	O	0.788737	4.135934	-1.651769

(continued 1).

No.	Symbol	X	Y	Z
71	O	0.221194	6.83089	-1.166585
72	H	-1.177325	2.771566	-2.025671
73	C	-2.422825	2.598961	-3.743007
74	H	0.953617	5.342873	-3.339892
75	C	-0.593627	5.999909	-1.955316
76	C	-1.678311	5.330872	-1.117253
77	O	-1.492049	1.735587	-4.368196
78	H	-1.894108	6.983214	-0.142209
79	H	-3.191128	2.0327	-3.201594
80	H	-2.906124	3.252778	-4.481884
81	H	-1.942507	0.911069	-4.607187
82	O	4.370376	4.2398	-2.300063
83	C	4.616128	2.851338	-2.176189
84	C	2.082397	3.59254	-1.835886
85	C	3.169385	4.662988	-1.660744
86	H	2.165732	2.853323	-1.026283
87	H	2.501942	3.562009	-3.969726
88	O	1.233882	1.999916	-3.486006
89	H	3.27404	1.158153	-2.317768
90	O	5.867876	2.609876	-2.731929
91	O	4.028267	1.542714	-4.206531
92	H	2.854218	5.571407	-2.188395
93	C	3.416694	5.011216	-0.191285
94	H	4.599548	2.569957	-1.10966
95	C	3.565963	2.007032	-2.958264
96	C	2.306599	2.848093	-3.157188
97	O	2.215001	5.443981	0.437664
98	H	0.496336	2.537019	-3.826445
99	H	6.497065	3.243177	-2.358537
100	H	4.994884	1.437568	-4.166493
101	H	3.849814	4.153261	0.341432
102	H	4.12873	5.837613	-0.133608
103	H	1.700078	4.658914	0.674214
104	H	0.821328	6.277579	-0.628818
105	O	-6.631474	3.563514	0.528216
106	C	-7.248654	2.700093	-0.387649
107	C	-4.763131	4.014996	-1.021757
108	C	-5.209635	3.621623	0.399954
109	H	-4.890779	5.097952	-1.173923
110	H	-5.247143	2.209392	-2.069224
111	O	-5.312493	3.84704	-3.360264
112	H	-7.378477	4.355145	-1.715674
113	O	-6.656835	1.428329	-0.285245
114	O	-7.802411	2.603211	-2.730153
115	H	-4.786224	2.634084	0.602885
116	C	-4.787762	4.604352	1.49174
117	H	-8.312443	2.656438	-0.12467
118	C	-7.04652	3.307877	-1.773294
119	C	-5.558809	3.26163	-2.084675
120	O	-3.398966	4.72552	1.658507
121	H	-5.161834	3.1417	-3.999456
122	H	-5.262855	5.574542	1.2797
123	H	-5.203788	4.232944	2.434473
124	H	-3.065068	5.281047	0.93305
125	O	-5.858774	-1.461197	0.419291
126	C	-6.1124	-2.179081	-0.750897
127	C	-7.48934	0.284237	-0.232168
128	C	-6.95739	-0.66561	0.858097
129	H	-8.513368	0.581139	0.036119
130	H	-7.653142	0.414039	-2.360866
131	O	-8.703504	-1.221475	-1.58792
132	H	-6.503837	-1.785315	-2.84144
133	O	-5.000358	-3.045433	-0.892292
134	O	-5.212558	-0.345978	-2.086677
135	H	-6.536106	-0.048153	1.658189
136	C	-8.074873	-1.520543	1.45269
137	H	-7.019642	-2.796875	-0.650143
138	C	-6.305448	-1.212839	-1.923508
139	C	-7.558461	-0.38056	-1.615412
140	O	-7.492496	-2.3725	2.417704

(continued 2).

No.	Symbol	X	Y	Z
141	H	-8.855999	-1.560057	-2.478828
142	H	-4.50003	-0.787131	-2.576558
143	H	-8.807661	-0.836263	1.903835
144	H	-8.592922	-2.08588	0.668758
145	H	-8.192323	-2.925001	2.783565
146	H	-7.576492	2.976623	-3.59273
147	O	6.589485	-2.382763	-1.162601
148	C	7.06337	-1.251324	-1.840279
149	C	4.49272	-2.538521	-2.269148
150	C	5.187185	-2.389962	-0.922657
151	H	4.837449	-3.458547	-2.760673
152	O	3.098296	-2.581538	-2.052207
153	H	4.479792	-0.441508	-2.514403
154	O	4.256613	-1.357895	-4.35519
155	H	6.76673	-1.914535	-3.867186
156	O	6.77633	-0.031725	-1.192678
157	O	6.687165	0.11948	-3.815656
158	H	4.873485	-1.434071	-0.478527
159	C	4.906206	-3.466814	0.095859
160	H	8.146349	-1.387454	-1.913702
161	C	6.383446	-1.123953	-3.212588
162	C	4.86435	-1.300589	-3.083862
163	O	5.499855	-3.036687	1.317343
164	H	4.020529	-0.447038	-4.591875
165	H	3.819171	-3.58417	0.192305
166	H	5.333337	-4.419826	-0.240283
167	H	5.281887	-3.680619	2.002046
168	O	8.689082	1.816329	0.360433
169	C	9.435546	1.049764	1.282491
170	C	6.900489	0.135676	0.216716
171	C	7.286566	1.608187	0.432942
172	H	5.89957	-0.022101	0.651372
173	H	7.753711	-1.839398	0.540771
174	O	7.394819	-0.873395	2.330671
175	H	9.828464	-1.011926	1.723232

No.	Symbol	X	Y	Z
176	O	10.776014	1.374185	1.095054
177	O	9.786486	-0.682893	-0.334596
178	H	6.87163	2.164115	-0.412308
179	C	6.678721	2.23024	1.694257
180	H	9.103369	1.256618	2.311826
181	C	9.291367	-0.428831	0.962025
182	C	7.8263	-0.844475	0.98076
183	O	5.314431	2.543936	1.463598
184	H	6.618346	-1.455391	2.339584
185	H	10.868008	2.333484	1.167688
186	H	10.544893	-0.094136	-0.460181
187	H	6.786637	1.575067	2.565347
188	H	7.202348	3.17029	1.8898
189	H	4.787351	1.753543	1.625573
190	H	6.695388	0.782114	-3.10292
191	O	0.353602	-3.301697	-4.31762
192	C	-0.146923	-4.517602	-3.819457
193	C	2.34881	-3.31543	-3.001936
194	C	1.056221	-2.566132	-3.310957
195	H	2.921456	-3.444306	-3.928584
196	H	1.429489	-4.488249	-1.474338
197	O	3.134687	-5.422793	-2.197802
198	H	1.591922	-5.687032	-4.261382
199	O	-0.992871	-4.313479	-2.721267
200	O	0.55692	-6.56178	-2.683351
201	H	0.437183	-2.536464	-2.407511
202	C	1.231715	-1.140707	-3.772027
203	H	-0.696539	-4.984909	-4.644513
204	C	1.019046	-5.408101	-3.368239
205	C	1.962132	-4.670272	-2.421675
206	O	-0.025967	-0.495592	-3.700145
207	H	2.85205	-6.284486	-1.861882
208	H	1.957413	-0.65419	-3.10883
209	H	1.632156	-1.121817	-4.794283
210	H	0.148027	0.452754	-3.594159

(continued 3).

No.	Symbol	X	Y	Z
211	O	-4.467568	-3.214751	-3.136979
212	C	-5.076175	-3.850367	-2.042396
213	C	-2.396207	-4.384056	-2.914471
214	C	-3.060283	-3.011289	-2.977723
215	H	-2.629442	-4.956345	-3.824455
216	H	-2.625761	-4.544198	-0.802896
217	O	-2.380399	-6.428031	-1.641581
218	H	-4.721714	-5.907772	-2.523052
219	O	-4.981495	-5.719056	-0.526042
220	H	-2.872274	-2.498543	-2.022851
221	C	-2.616712	-2.082993	-4.095433
222	H	-6.122878	-4.00484	-2.329452
223	C	-4.441301	-5.213595	-1.722893
224	C	-2.920568	-5.130424	-1.691042
225	O	-2.779575	-0.724328	-3.685022
226	H	-1.423027	-6.337516	-1.793707
227	H	-4.915759	-5.00032	0.120814
228	H	-1.573711	-2.262323	-4.36255
229	H	-3.251491	-2.248931	-4.969957
230	H	-1.952871	-0.513439	-3.215083
231	H	0.376223	-7.260266	-3.324593

Table S9. Cartesian coordinates of the optimized structure of C_{60}/trehalose.

No.	Symbol	X	Y	Z
1	C	1.355134	-2.504506	-2.301828
2	C	2.623170	-2.219036	-2.787849
3	C	1.201914	-3.215068	-1.045118
4	C	2.899131	-0.922240	-3.377337
5	C	0.305161	-1.507018	-2.384642
6	C	3.650437	-3.308223	-0.837471
7	C	3.796452	-2.629755	-2.038847
8	C	2.324037	-3.606785	-0.329273
9	C	1.895267	0.032689	-3.455129
10	C	0.569917	-0.266350	-2.946822
11	C	3.697126	-3.067269	1.497898
12	C	3.888028	-2.158659	2.528844
13	C	2.353030	-3.457819	1.114237
14	C	4.889469	-1.116013	2.401304
15	C	4.498895	-2.974886	0.291629
16	C	1.458584	-1.972194	2.857055
17	C	2.743672	-1.598842	3.223810
18	C	1.258804	-2.922800	1.778632
19	C	5.655982	-1.027737	1.248311
20	C	5.456324	-1.978030	0.169701
21	C	1.233371	2.892804	-1.429640
22	C	1.043415	1.983937	-2.461251
23	C	2.578276	3.280072	-1.045333
24	C	0.042926	0.938544	-2.332158
25	C	0.431987	2.799198	-0.221731
26	C	3.473942	1.795397	-2.787755
27	C	2.188437	1.422303	-3.153917
28	C	3.673113	2.745180	-1.709515
29	C	-0.714843	0.848436	-1.176095
30	C	-0.520532	1.800266	-0.099948
31	C	-0.439607	-1.306934	1.645896
32	C	-0.970038	-0.297421	0.854275
33	C	0.084680	-2.513080	1.030224
34	C	-0.676135	1.092965	1.156399
35	C	0.408726	-0.973813	2.775716
36	C	-0.496119	-1.599172	-1.177659
37	C	-0.996929	-0.446738	-0.589390
38	C	0.056253	-2.656615	-0.350430
39	C	0.134810	1.410250	2.236574
40	C	0.689774	0.353939	3.063708
41	C	5.372300	1.129266	-1.576121
42	C	5.903164	0.120279	-0.785264
43	C	4.846507	2.333601	-0.960389
44	C	5.609272	-1.268544	-1.087000
45	C	4.523811	0.796192	-2.705354
46	C	5.429147	1.420410	1.246633
47	C	5.932121	0.269079	0.658053
48	C	4.874654	2.475976	0.419321
49	C	4.797851	-1.586987	-2.166388
50	C	4.243204	-0.531525	-2.993678
51	C	3.577559	2.326682	2.371202
52	C	2.309523	2.041374	2.856892
53	C	3.729953	3.035736	1.114269
54	C	2.033616	0.744387	3.447268
55	C	4.627437	1.327963	2.452945
56	C	1.282159	3.129857	0.906897
57	C	1.135965	2.453486	2.109765
58	C	2.608066	3.428526	0.398562
59	C	3.037586	-0.210177	3.525514
60	C	4.363993	0.088301	3.017568
61	C	-3.425949	-3.093755	-0.017046
62	C	-4.784173	-2.980635	0.684423
63	C	-5.954908	-3.559817	-0.110488
64	C	-6.450449	-2.606212	-1.216211
65	C	-6.639520	-1.186852	-0.658775
66	C	-5.333982	-0.768523	0.004265
67	O	-5.056368	-1.642073	1.088455
68	O	-5.458640	0.515009	0.535770
69	C	-4.206884	1.077293	0.853070
70	C	-4.427022	2.343966	1.675508

(continued).

No.	Symbol	X	Y	Z
71	C	-5.054825	3.435267	0.821126
72	C	-4.197690	3.647297	-0.411379
73	C	-4.099175	2.324343	-1.168327
74	O	-3.470138	1.366689	-0.311356
75	C	-3.306413	2.453653	-2.455317
76	O	-2.217912	3.337607	-2.245529
77	O	-4.787798	4.666002	-1.193082
78	O	-5.123521	4.594988	1.624994
79	O	-5.216728	2.043116	2.799736
80	O	-7.718563	-1.126646	0.241667
81	O	-5.517782	-2.505055	-2.274355
82	O	-6.995971	-3.775261	0.837529
83	O	-3.036357	-4.444085	-0.164189
84	H	-2.704439	-2.515307	0.575705
85	H	-3.460155	-2.674627	-1.024214
86	H	-4.725546	-3.550217	1.619290
87	H	-5.627175	-4.508718	-0.557501
88	H	-7.426156	-2.963133	-1.576852
89	H	-6.854064	-0.505209	-1.488119
90	H	-4.514003	-0.794526	-0.727654
91	H	-3.603986	0.351422	1.410297
92	H	-3.426074	2.693961	1.976348
93	H	-6.062101	3.115153	0.511101
94	H	-3.186351	3.932083	-0.092168
95	H	-5.107870	1.970646	-1.423270
96	H	-3.976326	2.843778	-3.231775
97	H	-2.968411	1.454628	-2.758690
98	H	-1.689045	3.363081	-3.051782
99	H	-4.100717	4.994945	-1.788418
100	H	-5.423297	5.313059	1.051074
101	H	-5.415000	2.890091	3.222392
102	H	-7.566135	-1.826018	0.897130
103	H	-5.417789	-3.373753	-2.684154
104	H	-7.734210	-4.212376	0.393053
105	H	-2.830557	-4.794572	0.711343

Table S10. Cartesian coordinates of the optimized structure of C_{60}/sucrose.

No.	Symbol	X	Y	Z
1	C	4.009782	-2.993689	1.418149
2	C	3.182152	-2.589072	2.455237
3	C	3.435349	-3.496151	0.183403
4	C	3.490433	-1.383761	3.20207
5	C	5.185049	-2.212048	1.080383
6	C	1.191706	-3.148322	1.124671
7	C	1.740807	-2.666123	2.304109
8	C	2.057748	-3.571989	0.039772
9	C	4.613061	-0.635945	2.878583
10	C	5.479474	-1.059409	1.794035
11	C	0.185322	-2.51105	-0.90012
12	C	-0.227027	-1.421068	-1.653837
13	C	1.4359	-3.178442	-1.211149
14	C	-0.810432	-0.264241	-0.999607
15	C	0.036217	-2.492036	0.54349
16	C	1.787551	-1.586254	-3.051567
17	C	0.591485	-0.948912	-2.755211
18	C	2.21912	-2.72622	-2.263016
19	C	-0.956596	-0.247035	0.380209
20	C	-0.524034	-1.386048	1.170587
21	C	4.944615	2.37402	0.997091
22	C	5.356758	1.285148	1.751475
23	C	3.693784	3.04056	1.308774
24	C	5.939299	0.12789	1.097571
25	C	5.095908	2.355897	-0.446139
26	C	3.341964	1.450656	3.150572
27	C	4.537424	0.813139	2.852422
28	C	2.910854	2.589712	2.36153
29	C	6.083762	0.110378	-0.282066
30	C	5.652797	1.249497	-1.071272
31	C	4.119802	-1.460259	-2.809352
32	C	5.15353	-0.701631	-2.279497
33	C	3.660306	-2.647636	-2.112773
34	C	5.077758	0.747486	-2.305625
35	C	2.962551	-0.804594	-3.389816
36	C	5.336134	-2.230297	-0.362869
37	C	5.775407	-1.095351	-1.028849
38	C	4.255001	-3.02403	-0.917064
39	C	3.971742	1.37417	-2.861102
40	C	2.890039	0.580616	-3.414888
41	C	1.009836	1.323692	2.907411
42	C	-0.025416	0.56525	2.379769
43	C	1.469487	2.510646	2.210541
44	C	0.051184	-0.883206	2.405739
45	C	2.167047	0.66841	3.488401
46	C	-0.206522	2.095463	0.461258
47	C	-0.647456	0.958893	1.12883
48	C	0.875024	2.888557	1.015359
49	C	1.158063	-1.510154	2.959628
50	C	2.239572	-0.71671	3.513515
51	C	1.120288	2.85857	-1.320246
52	C	1.948126	2.452901	-2.35666
53	C	1.694447	3.360441	-0.085686
54	C	1.639245	1.247454	-3.103587
55	C	-0.053538	2.075829	-0.982683
56	C	3.938506	3.011087	-1.026713
57	C	3.389263	2.531162	-2.206861
58	C	3.07209	3.434735	0.057815
59	C	0.515925	0.49998	-2.781416
60	C	-0.350203	0.923501	-1.696588
61	C	-6.081731	2.464663	1.903857
62	C	-8.04515	-1.376623	-0.12079
63	C	-3.482652	-2.464602	-1.1583
64	C	-5.089513	1.878771	0.914799
65	C	-6.793371	-2.169748	0.167476
66	C	-4.218143	2.962371	0.267202
67	C	-6.075424	-1.739835	1.441493
68	C	-3.417897	2.375969	-0.88512
69	C	-4.321093	1.615195	-1.843294
70	C	-4.630554	-2.073573	1.108718

(continued).

No.	Symbol	X	Y	Z
71	C	-5.134041	0.571827	-1.077496
72	C	-4.559563	-1.713664	-0.384716
73	O	-6.993919	3.330214	1.258818
74	O	-8.657292	-1.921825	-1.272257
75	O	-3.29974	-1.940052	-2.446406
76	O	-3.381718	3.565625	1.2336
77	O	-6.601521	-2.432491	2.545585
78	O	-2.734714	3.410797	-1.553014
79	O	-3.474156	1.010547	-2.809796
80	O	-3.755175	-1.367982	1.946871
81	O	-5.887777	1.216277	-0.07281
82	O	-5.831054	-2.008403	-0.892106
83	O	-4.221708	-0.331139	-0.528845
84	H	-4.442699	1.14266	1.417177
85	H	-7.047578	-3.235453	0.241735
86	H	-4.87245	3.754661	-0.114224
87	H	-6.157965	-0.649855	1.568861
88	H	-2.709751	1.643148	-0.468137
89	H	-5.015413	2.319553	-2.316949
90	H	-4.502032	-3.162306	1.207835
91	H	-5.85724	0.065757	-1.720387
92	H	-5.538743	3.050476	2.649134
93	H	-6.605065	1.64485	2.415976
94	H	-7.773797	-0.321275	-0.257413
95	H	-8.694001	-1.453169	0.764383
96	H	-3.779944	-3.512742	-1.250547
97	H	-2.555758	-2.424859	-0.563865
98	H	-7.362594	2.828305	0.518897
99	H	-9.437237	-1.393159	-1.475083
100	H	-3.178887	-0.978202	-2.352166
101	H	-2.716598	2.914216	1.498379
102	H	-6.148841	-2.107927	3.33431
103	H	-2.293133	2.994319	-2.307111
104	H	-3.953489	0.926104	-3.643702
105	H	-2.876632	-1.766533	1.895963

Table S11. Cartesian coordinates of the optimized structure of C_{60}/maltose.

No.	Symbol	X	Y	Z	No.	Symbol	X	Y	Z
1	C	2.996115	-0.993644	-3.372738	36	C	1.818706	-2.728953	-2.316867
2	C	3.172514	0.382082	-3.410455	37	C	0.869486	-3.012861	-1.345477
3	C	3.957347	-1.829879	-2.677297	38	C	3.229731	-2.902751	-2.025069
4	C	2.035394	1.265076	-3.227783	39	C	0.916215	-2.488748	2.180098
5	C	1.674378	-1.549493	-3.149965	40	C	2.327183	-2.661951	2.472507
6	C	5.237086	0.1858	-2.090071	41	C	2.170306	3.399677	-0.227471
7	C	4.318373	0.984993	-2.75495	42	C	3.519442	3.234566	0.051721
8	C	5.052378	-1.253122	-2.050342	43	C	1.177131	3.103487	0.78812
9	C	0.771385	0.733632	-3.015139	44	C	4.397949	2.641544	-0.939753
10	C	0.587045	-0.705349	-2.975177	45	C	1.638666	2.980579	-1.511121
11	C	5.913365	-0.574346	0.026229	46	C	2.987075	2.4803	2.330068
12	C	5.640974	-0.501807	1.384649	47	C	3.937077	2.76483	1.359546
13	C	5.47022	-1.722827	-0.742359	48	C	1.576423	2.654072	2.038363
14	C	5.212131	0.753789	1.97286	49	C	3.88953	2.24061	-2.166833
15	C	5.76925	0.605348	-0.806714	50	C	2.478509	2.413554	-2.458825
16	C	4.489772	-2.67282	1.302548	51	C	1.809848	0.744675	3.385978
17	C	4.912987	-1.574526	2.037047	52	C	1.633175	-0.630568	3.423845
18	C	4.774407	-2.748327	-0.118551	53	C	0.847895	1.580295	2.689459
19	C	5.074135	1.881467	1.176645	54	C	2.77013	-1.513151	3.241034
20	C	5.358954	1.805431	-0.244438	55	C	3.131315	1.300232	3.163023
21	C	-1.10868	0.326716	-0.011382	56	C	-0.431519	-0.43444	2.104256
22	C	-0.836026	0.254388	-1.370903	57	C	0.486255	-1.233292	2.768549
23	C	-0.665848	1.474811	0.757787	58	C	-0.247155	1.004241	2.064009
24	C	-0.405222	-1.002586	-1.959347	59	C	4.034421	-0.981859	3.028699
25	C	-0.968845	-0.855783	0.822963	60	C	4.219222	0.456672	2.988976
26	C	0.316842	2.424457	-1.288147	61	O	-5.740197	0.813289	0.142436
27	C	-0.107808	1.326652	-2.024435	62	C	-4.546448	1.587434	0.054342
28	C	0.030935	2.499865	0.133395	63	C	-4.562582	2.495838	-1.157034
29	C	-0.265783	-2.129041	-1.162083	64	O	-4.572435	1.698286	-2.32278
30	C	-0.553017	-2.053502	0.259584	65	C	-3.306076	3.354769	-1.119826
31	C	2.635515	-3.648582	0.241884	66	O	-3.353164	4.20126	-2.247927
32	C	1.286637	-3.482728	-0.036968	67	C	-3.211577	4.11079	0.20639
33	C	3.628515	-3.351144	-0.774117	68	O	-3.231323	3.19416	1.27953
34	C	0.406681	-2.891542	0.954301	69	C	-4.409677	2.39501	1.343328
35	C	3.167559	-3.228733	1.525098	70	C	-4.296677	1.488316	2.560989

(continued).

No.	Symbol	X	Y	Z
71	O	-3.277272	0.522153	2.431764
72	C	-5.598854	-0.500126	-0.293937
73	O	-4.676967	-1.138718	0.578422
74	C	-4.38727	-2.485505	0.212815
75	C	-5.658566	-3.333119	0.242621
76	O	-5.962076	-3.539976	1.605697
77	C	-6.829851	-2.662685	-0.498731
78	O	-6.590708	-2.843509	-1.882308
79	C	-6.963012	-1.167095	-0.18586
80	O	-7.488833	-1.015259	1.109355
81	C	-3.615397	-2.559191	-1.104066
82	O	-2.902252	-3.781079	-1.072626
83	H	-3.678802	0.921302	-0.026738
84	H	-5.447615	3.148585	-1.11811
85	H	-4.38214	2.298429	-3.058078
86	H	-2.443905	2.676302	-1.172917
87	H	-2.454998	4.473885	-2.470824
88	H	-5.293158	3.038793	1.460925
89	H	-5.273316	1.018658	2.732798
90	H	-4.057652	2.109302	3.429325
91	H	-3.623904	-0.180216	1.857068
92	H	-5.222665	-0.531397	-1.325769
93	H	-3.718422	-2.858398	0.995488
94	H	-5.444806	-4.280609	-0.27344
95	H	-6.806408	-4.003169	1.665637
96	H	-7.761803	-3.158287	-0.188787
97	H	-7.360987	-2.530679	-2.372878
98	H	-7.623388	-0.710616	-0.940884
99	H	-7.369537	-0.085618	1.349283
100	H	-2.94186	-1.689699	-1.139371
101	H	-4.286108	-2.516803	-1.967592
102	H	-2.517422	-3.928017	-1.944482
103	H	-2.243149	4.620255	0.291434
104	O	-4.274976	5.014415	0.263334
105	H	-4.170461	5.548285	1.061747

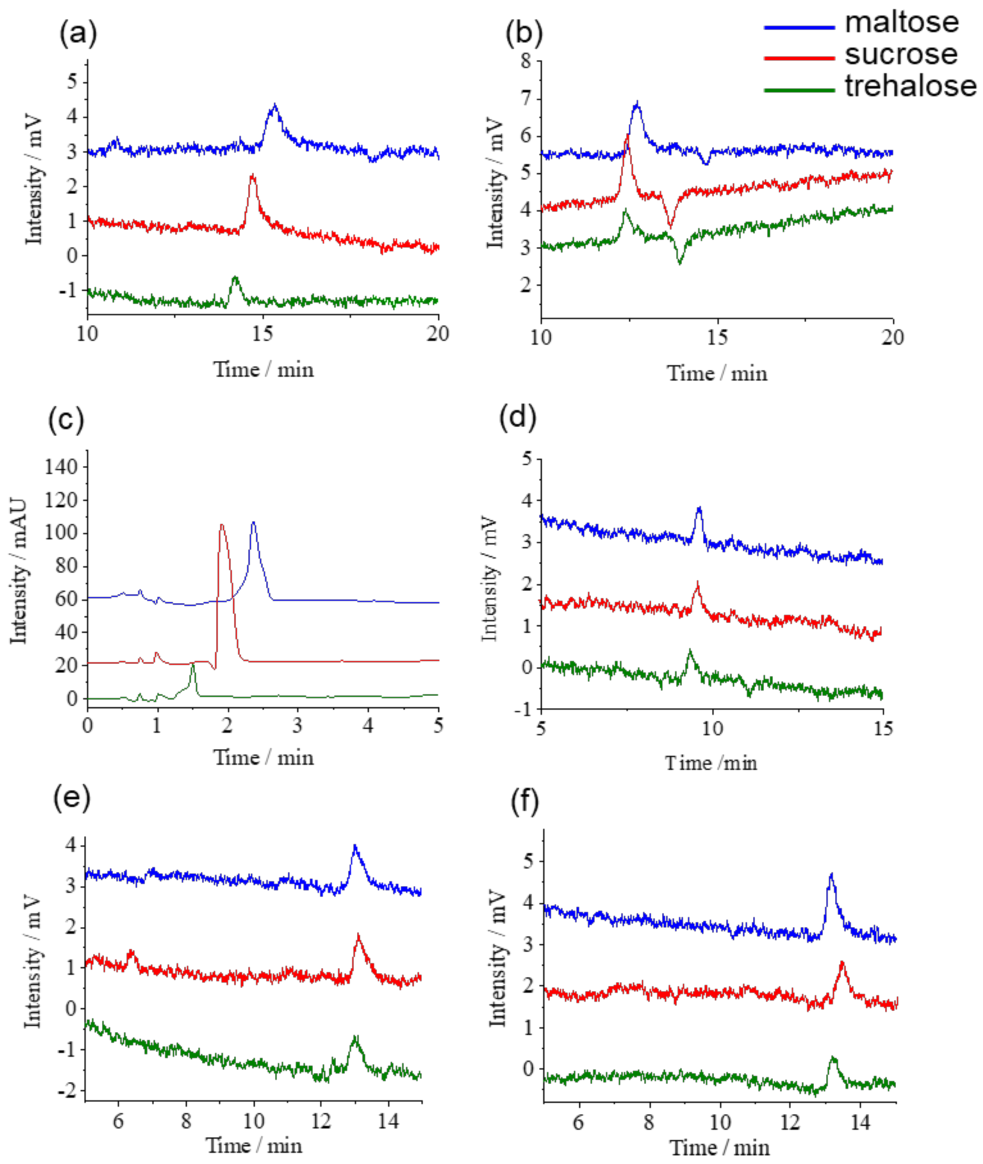

Figure S12. Chromatograms of di-saccharides.
(a) C60, (b) C70, (c) PGC, (d) ODS, (e) Silica, (f) Amino
