# Supplementary material for: Separation of saccharides using fullerene-bonded silica monolithic columns via π interactions in liquid chromatography
Source: Sci Rep. 2020 Aug 14;10:13850. doi: 10.1038/s41598-020-70904-3 (PMC7429847; doi:10.1038/s41598-020-70904-3)
Supplement: Supplementary file 2 — Supplementary Information 2. [file 41598_2020_70904_MOESM2_ESM.docx]

**Supplementary Information**

Structures of the analytes, synthesis of NHS-PFPA-C70 and NHS-PFPA-C60, the reparation of a C70 column and C60 column, and FRIR spectra of the synthesized compounds, separation of 2AB-Glcs with ODS-silica monolithic capillary, values of *E*_binding_, molecular geometry of the complexes, cartesian coordinates of the optimized structures, and chromatograms of di-saccharides.
